# Single-cell and bulk transcriptional profiling of mouse ovaries reveals novel genes and pathways associated with DNA damage response in oocytes

**DOI:** 10.1016/j.ydbio.2024.09.007

**Published:** 2024-09-19

**Authors:** Monique Mills, Chihiro Emori, Parveen Kumar, Zachary Boucher, Joshy George, Ewelina Bolcun-Filas

**Affiliations:** a The Jackson Laboratory, 600 Main Street, Bar Harbor, ME, 04609, USA; b The Graduate School of Biomedical Science and Engineering, University of Maine, Orono, ME, 04469, USA; c The Jackson Laboratory for Genomic Medicine, Farmington, CT, 06110, USA; d Department of Experimental Genome Research, Research Institute for Microbial Diseases, Osaka University, Suita, Osaka, 5650871, Japan

**Keywords:** DNA damage response, CHEK2, Oocyte, Ovary, Single-cell transcriptomics

## Abstract

Immature oocytes enclosed in primordial follicles stored in female ovaries are under constant threat of DNA damage induced by endogenous and exogenous factors. Checkpoint kinase 2 (CHEK2) is a key mediator of the DNA damage response (DDR) in all cells. Genetic studies have shown that CHEK2 and its downstream targets, p53, and TAp63, regulate primordial follicle elimination in response to DNA damage. However, the mechanism leading to their demise is still poorly characterized. Single-cell and bulk RNA sequencing were used to determine the DDR in wild-type and *Chek2*-deficient ovaries. A low but oocyte-lethal dose of ionizing radiation induces ovarian DDR that is solely dependent on CHEK2. DNA damage activates multiple response pathways related to apoptosis, p53, interferon signaling, inflammation, cell adhesion, and intercellular communication. These pathways are differentially employed by different ovarian cell types, with oocytes disproportionately affected by radiation. Novel genes and pathways are induced by radiation specifically in oocytes, shedding light on their sensitivity to DNA damage, and implicating a coordinated response between oocytes and pregranulosa cells within the follicle. These findings provide a foundation for future studies on the specific mechanisms regulating oocyte survival in the context of aging, therapeutic and environmental genotoxic exposures.

## 1. Introduction

DNA damage response (DDR) is essential in cell and organ function. DNA damage triggers the DDR, a complex network of cellular pathways that detect, signal, repair the damage, and trigger apoptosis if damage is irreparable. However, the response mechanisms and cellular outcomes of DNA damage may differ depending on the cell type, including cell death, senescence, or terminal differentiation (Blanpain et al., 2011; Cinat et al., 2021; Fortini et al., 2013; Hoch, 2023). DNA damage encountered by cells via endogenous and exogenous mechanisms is also implicated in cellular and organ aging (d’Adda di Fagagna, 2008; Schumacher et al., 2021; Yousefzadeh et al., 2021). The ovaries are organs that naturally age early due to a lack of regenerative abilities, resulting in the cessation of estrogen production and menopause at the average age of 50–51 (te Velde and Pearson, 2002). Estrogen is produced by growing follicles recruited from the primordial follicle (PF) pool. Each female is born with a finite number of PFs, each containing an immature oocyte surrounded by somatic cells (primordial oocyte). As a woman ages, the PF reserve gradually diminishes, and the remaining PFs become more susceptible to DNA damage and other forms of cellular stress.

Endogenous DNA damage may arise during normal cellular activities such as replication and transcription or from reactive oxygen species (ROS) produced during normal metabolic processes. Exogenous DNA damage can be caused by environmental exposures such as ionizing radiation (IR), alkylating agents, heavy metals, and various chemicals. For ovaries, it has been shown that IR- and chemotherapy-induced DNA damage causes accelerated depletion of the ovarian follicle reserve (Nguyen et al., 2019; Spears et al., 2019). Multiple studies have highlighted the critical role of checkpoint kinase 2 (CHEK2) in establishing and maintaining the ovarian follicle reserve (Bolcun-Filas et al., 2014; Emori et al., 2023; Martínez-Marchal et al., 2020; Powers et al., 2020; Rinaldi et al., 2017). Moreover, *CHEK2* loss-of-function variants in humans correlate with females having a larger ovarian follicle reserve and later onset of menopause (Ruth et al., 2021; Ward et al., 2022). These indicate that CHEK2 signaling in the ovary regulates follicle survival during women’s reproductive life not only in response to genotoxic insults but also in response to endogenous sources of DNA damage. Improper repair of DNA damage can lead to mutations and genomic rearrangements. Therefore, cells have evolved a DDR and quality checkpoints that ensure DNA fidelity before cells can divide. DNA double-strand breaks (DSBs) are the most lethal type of DNA damage. Cells respond to DSBs by activating a multi-layered signaling cascade involving many proteins and processes, and CHEK2 is a key checkpoint kinase mediating DDR (Lanz et al., 2019). CHEK2 phosphorylates and activates TRP53 (henceforth p53), which in turn plays a critical role in coordinating DDR outcomes and cell fate decisions, such as cell cycle arrest or apoptosis. Cell cycle arrest allows more time to repair DSBs, after which cells can re-enter the cell cycle. If DNA damage persists unrepaired, p53 triggers apoptosis. In contrast to somatic cells, which rely mainly on p53 activity, oocytes express a related protein TRP63 (p63) (Livera et al., 2008; Suh et al., 2006). TA isoform of p63 (henceforth TAp63) expressed exclusively in oocytes in the ovary has been shown to trigger oocyte apoptosis after activation by CHEK2 in response to DNA damage (Bolcun-Filas et al., 2014; Emori et al., 2023; Tuppi et al., 2018). Animal studies show that the inactivation of CHEK2 or TAp63, but not p53, prevents apoptotic elimination of oocytes exposed to low doses of IR (0.2–0.5 Gy) (Bolcun-Filas et al., 2014; Kim et al., 2019; Livera et al., 2008; Suh et al., 2006). This indicates that for primordial oocytes the primary response to DSBs appears to be an imminent apoptotic elimination—even though continuous meiotic arrest (months in mice and decades in humans) could allow for efficient repair. However, our knowledge of DDR in primordial oocytes is still minimal compared to somatic cells or fully grown oocytes and is primarily based on the activities of the few aforementioned proteins (Carroll and Marangos, 2013; Gebel et al., 2020; Pailas et al., 2022).

To further our understanding of the mechanism regulating primordial oocyte survival or death in response to genotoxic insults, we conducted bulk and single-cell transcriptomic analysis of ovarian response to DNA damage in wild-type and CHEK2-deficient females. To model DNA damage, we employed IR, which is known to induce DSBs and apoptosis in primordial oocytes. We show that—in addition to apoptosis—DNA damage activates multiple response pathways in the ovary related to p53, interferon signaling, inflammation, cell adhesion, and intercellular communication. Our results indicate that different cell types within the ovary employ these pathways differently and that oocytes mount the strongest cellular response. We identified novel genes involved in oocyte-specific DDR that may participate in pro-survival response or contribute to primordial oocyte sensitivity to DNA damage. Considering the importance of DNA damage in the process of aging, exposure to chemicals, and genotoxic therapies, our findings could pave the way for treatment strategies aimed at delaying ovarian aging and mitigating the toxic side effects of anti-cancer treatments.

## 2. Results

### 2.1. Radiation-induced DNA damage in ovaries activates CHEK2-dependent signaling

**Fig. 1.**
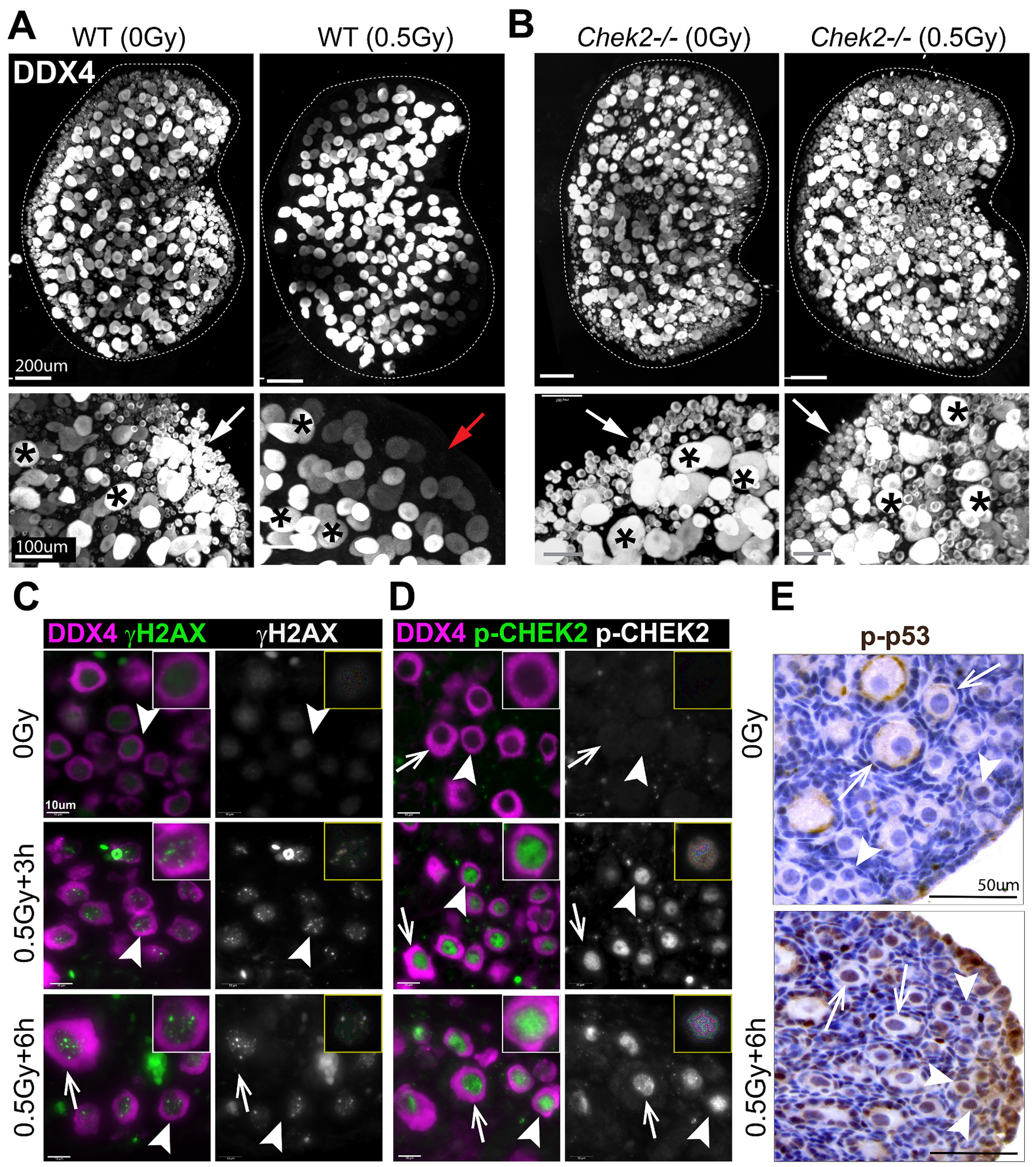
IR-induced DNA damage activates CHEK2-dependent signaling leading to primordial oocyte loss. IR dose of 0.5 Gy eliminates primordial oocytes (white arrows) in wild-type (**A**) but not in *Chek2*-deficient ovaries (**B**). Large growing oocytes (asterisks) are not eliminated by IR. The red arrow indicates the area depleted of primordial oocytes in wild-type irradiated ovaries. Shown are 3D renderings of whole ovaries (top) and a magnified view (bottom). Ovaries were collected one week after IR and immunostained with oocyte marker DDX4. IR induces DNA damage in oocytes, as evidenced by γH2AX staining (**C**), and leads to phosphorylation and activation of CHEK2 (p-CHEK2) (**D**). IR leads to phosphorylation and activation of p53 (p-p53) in oocytes and other ovarian cells (**E**). Arrowheads indicate primordial oocytes and arrows indicate oocytes in primary follicles.

A single dose of relatively low radiation (≤ 0.5 Gy) eradicates primordial oocytes in mice within a few days (Puy et al., 2021). One week after IR, no primordial oocytes were observed in ovaries, while larger oocytes in growing follicles were still present (Fig. 1A). The elimination of primordial oocytes is dependent on CHEK2 as abundant primordial oocytes are present in *Chek2*^−/−^ ovaries one week after radiation (Fig. 1B) (Bolcun-Filas et al., 2014). IR induces DNA DSBs, which were readily detectable in ovaries 6 h after IR; all oocytes stained positive for DNA damage marker γH2AX (Fig. 1C). IR-induced damage activated CHEK2 as evidenced by the positive staining for phosphorylated CHEK2 at threonine 68 (pCHEK2) (Ward et al., 2001) (Fig. 1D). Similar to γH2AX, pCHEK2 was predominantly activated in oocytes (Fig. 1D). This suggests that IR-induced damage triggers a different response in oocytes than in pregranulosa cells or other ovarian somatic cells. CHEK2 phosphorylates p53 in response to DSBs, which prevents p53 degradation by an MDM2-dependent mechanism (Shi and Gu, 2012). Because p53 seems dispensable for oocyte apoptosis, we next tested p53 activation by IR in ovaries. Phosphorylated p53 (S15) was detected in oocytes, pregranulosa cells, and other cell types in irradiated ovaries (Fig. 1E). TAp63 is a direct target of CHEK2 phosphorylation and the key proapoptotic factor in oocytes (Bolcun-Filas et al., 2014; Emori et al., 2023; Livera et al., 2008; Luan et al., 2022). Therefore, activation of p53 in oocytes after IR suggests that p53 may still play a role in the DDR and potentially regulates other cellular responses in oocytes. To summarize, IR exposure induces DNA DSBs in oocytes—and to a lesser extent in somatic cells—leading to activation of CHEK2-dependent signaling through TAp63 and p53. In the absence of CHEK2, TAp63, and p53 fail to induce apoptosis, resulting in primordial oocyte survival.

### 2.2. Transcriptional response to radiation-induced DNA damage in ovaries is CHEK2 dependent

**Fig. 2.**
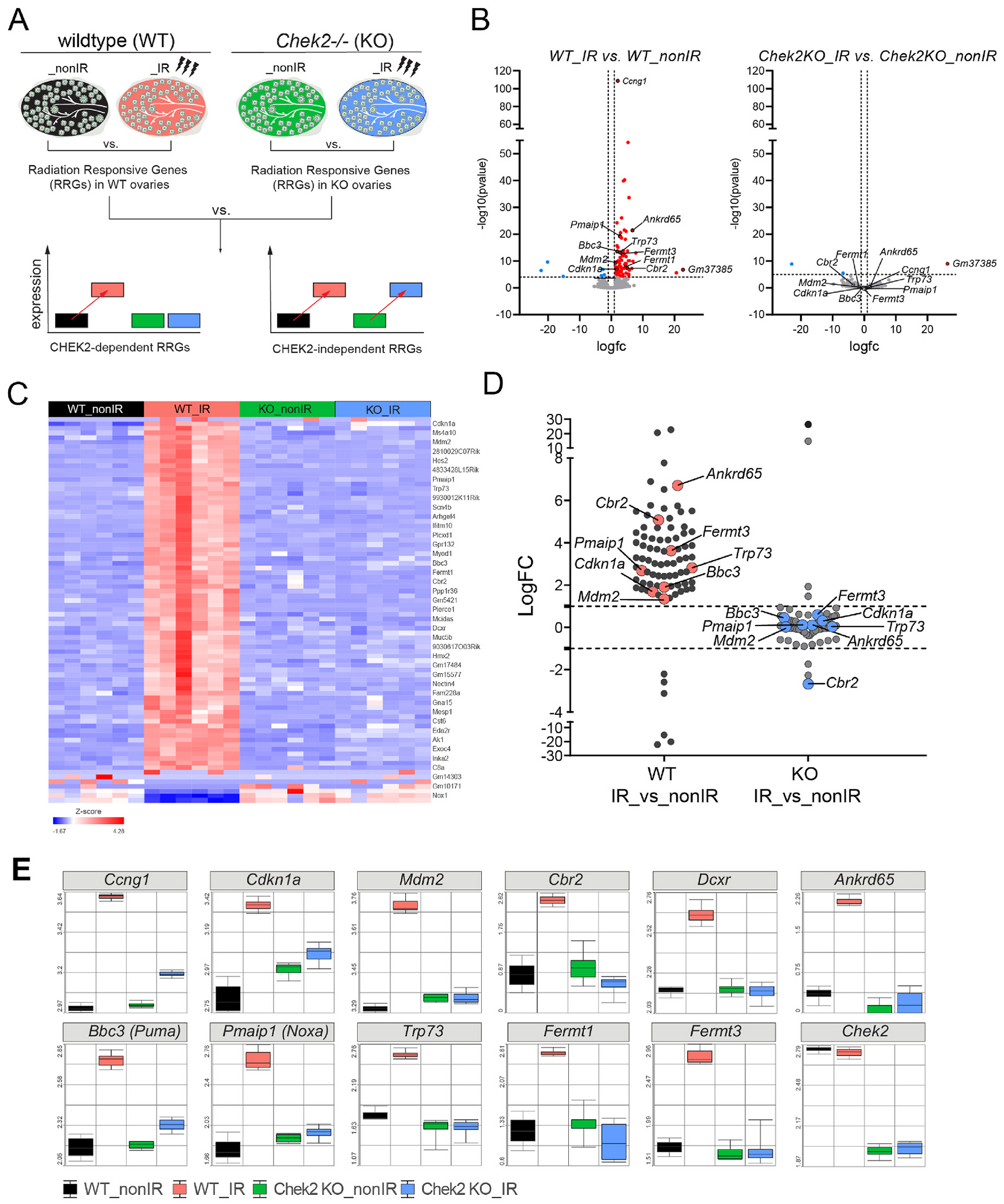
Radiation response in ovaries is abolished in the absence of CHEK2. **A)** Schematic representation of the experimental design. **B**) Volcano plot showing significant Radiation-Responsive Genes (RRGs) at FDR < 0.05 in wild-type and *Chek2*^*−/−*^ ovaries. Up-regulated LogFC ≥ 1 (red), non-significant (grey), and downregulated LogFC ≤ −1 (blue). **C**) Heatmap showing expression of 83 RRGs across treatment groups. **D**) Comparison of gene expression changes (LogFC) induced by IR in wild-type vs. *Chek2*^*−/−*^ ovaries shows a lack of response in the absence of CHEK2. **E**) Box plots showing expression of genes representing known DDR markers and newly identified RRGs in the ovary (N = 6 samples per group).

In addition to p53 and TAp63, CHEK2 also phosphorylates and activates other targets, including CDC25, NEK6, FOXM1, BRCA1, and BRCA2 during DDR (Chehab et al., 2000; Hirao et al., 2000; van Jaarsveld et al., 2020; Zannini et al., 2014). To identify proteins involved in the ovarian DDR signaling downstream of CHEK2, we performed transcriptional profiling of ovaries exposed to an oocyte-lethal dose of IR in wild-type and CHEK2-deficient females. We used juvenile ovaries for analysis because they are enriched for primordial oocytes that are extremely sensitive to DNA damage. Most primordial oocytes are depleted within 24 h after IR (Stringer et al., 2020). At 6 h post-IR, primordial oocytes positive for DDR markers remained in the ovary, suggesting active and ongoing response (Fig. 1C). We hypothesized that analysis of differentially expressed genes in wild-type (radiation sensitive) and *Chek2*^−/−^ ovaries (radiation resistant) would identify Radiation-Responsive Genes (RRGs) and signaling pathways downstream of CHEK2; those most likely contributing to primordial oocyte elimination (Fig. 2A). RRGs differentially expressed in both wild-type and *Chek2*^−/−^ ovaries will represent CHEK2-independent response pathways, which we consider less likely to contribute to primordial oocyte elimination. We exposed one-week-old wild-type and *Chek2*^−/−^ females to 0.5 Gy IR or sham treatment (N = 6 for each group) and collected ovaries 6 h post-IR for RNA extraction and subsequent bulk RNA sequencing. Differential gene expression analysis in wild-type ovaries identified 83 RRGs with ≥ 2-fold change in expression and FDR ≤ 0.05 (Supplementary Data 1). 77 genes were upregulated and 6 downregulated (Fig. 2B, C; Table S1). 70 RRGs were protein coding genes, 7 were lncRNAs, and 6 were unclassified or pseudogenes. Surprisingly, overall global gene expression was not significantly altered by IR in *Chek2*^−/−^ ovaries (Fig. 2B, C), and only 3 genes showed a significant change (≥ 2-fold change and FDR ≤ 0.05) (Table S1; Supplementary Data 1). Two of them are unclassified genes, while *Padi6*, a known component of cytoplasmic lattices in growing oocytes, has not been linked to DDR. Many RRGs were previously reported to participate in the p53 signaling pathway, apoptosis, or cell cycle (*Bbc3*, *Ccng1*, *Cdkn1a* (*p21*), *Trp73*, *Mdm2*, *Pmaip1, Tp53inp1, Lhx3, Eda2r, Nox1)* and were upregulated in response to IR in a CHEK2 dependent manner (Fig. 2D, E). These results confirm that CHEK2 is the major regulator of ovarian response to an oocyte-lethal dose of IR. In addition to known cell cycle arrest and proapoptotic genes, we detected CHEK2-dependent upregulation of genes with unknown roles in the ovary such as *Cbr2* (Carbonyl reductase 2; LogFC = 5.07; FDR = 2.91E-05), *Dcxr* (Dicarbonyl and L-xylulose reductase; LogFC = 1.5; FDR = 0.003), *Ankrd65* (Ankyrin repeat domain 65; LogFC = 6.7; FDR = 1.22E-18), *Fermt1* (LogFC = 4.5; FDR = 9.82E-06) and *Fermt3* (LogFC = 3.6; FDR = 4.15E-10) (Fig. 2D, E and Table S1). These results indicate that IR leads to CHEK2-dependent upregulation of known and novel genes in the ovary, which may play a role in DDR and regulation of primordial oocyte survival.

### 2.3. Radiation activates pathways related to apoptosis, interferon-mediated response, NF-κB signaling, and changes in apical junctions in the ovary

**Fig. 3.**
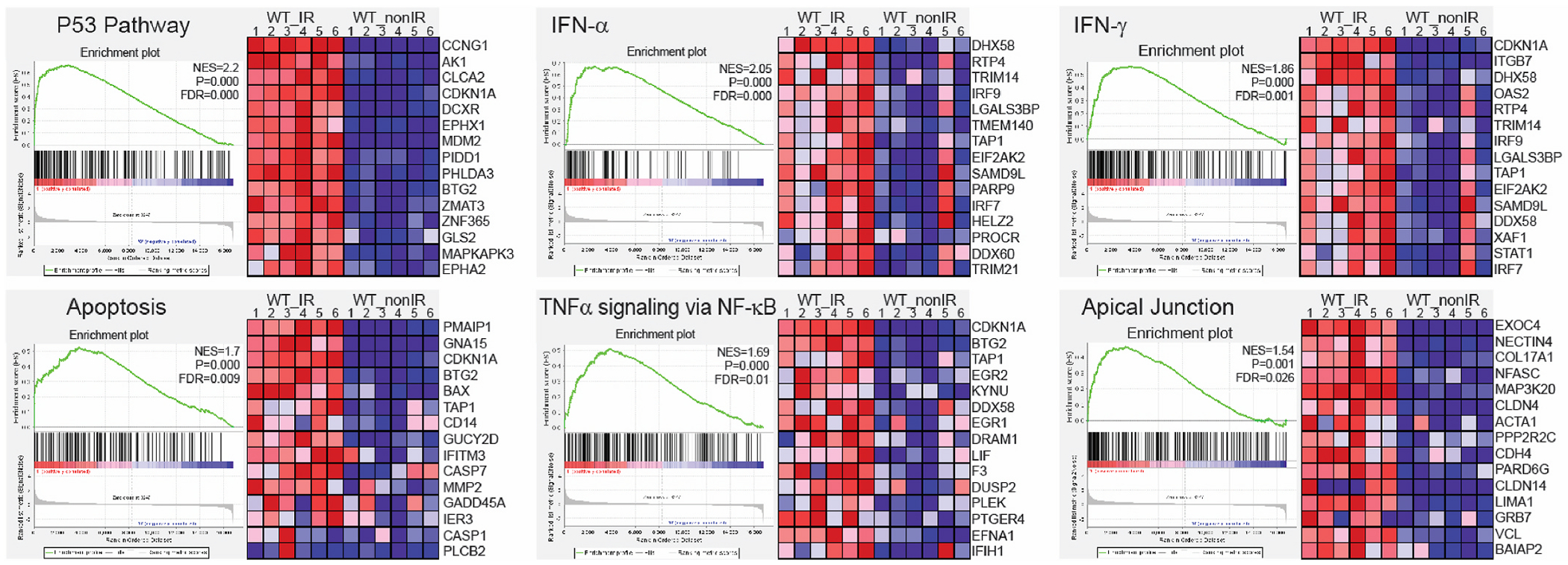
Gene Set Enrichment Analysis (GSEA) in HALLMARK datasets. The p53, apoptosis, inflammatory, and apical junctions gene sets were significantly activated in irradiated ovaries. The plots show the leading edge (most significant genes) as vertical bars accumulated below the peak of the green enrichment score (ES) line and on the right as heatmaps. NES - normalized ES, P - nominal p value, FDR - q value. The significance criteria were nominal p-value < 0.05 and FDR q-value ≤ 0.05.

To further identify the cellular and molecular response to IR in ovaries, we conducted a functional enrichment analysis for RRGs. The significantly enriched terms for Biological Process and KEGG pathways, as identified by g:Profiler (Raudvere et al., 2019), were associated with apoptosis and p53 activity (Table S2). These were driven by well-known genes such as *Pmaip1, Bbc3, Cdkn1a, Mdm2*, and *Trp73*. However, it is important to note that radiation responses are dynamic and vary among different cell types. As such, gene enrichment analysis based solely on differentially expressed genes with a ≥ 2-fold change might overlook significant effects on the activity of pathways involved in active responses. To address this, we conducted a Gene Set Enrichment Analysis (GSEA), which considers genes that are part of a differentially expressed set but may not individually reach statistical significance (Mootha et al., 2003; Subramanian et al., 2005). Cumulative small changes in the expression of multiple genes belonging to the same gene set (pathway), but in a coordinated manner, could reveal other pathways involved in the radiation response. We performed GSEA using the Molecular Signatures Database (MSigDB) and the Hallmark gene set collection, which includes curated and well-defined biological processes (Liberzon et al., 2011). GSEA revealed 12 gene sets with significant changes (FDR ≤ 0.05) in wild-type irradiated ovaries, including the activation of the p53 signaling and apoptosis pathways (Fig. 3 and Table S3). Furthermore, we detected activation of interferon-alpha (INF-α) and gamma (INF-γ) responses, as well as TNFα signaling via NF-κB. Activation of these pathways may suggest an inflammatory response to radiation in the ovary (Fig. 3 and Table S3). Inflammation is a recognized response to radiation-induced tissue damage and has been linked to anti-apoptotic signaling, cell death, and fibrosis (Antar et al., 2023; Di Maggio et al., 2015; Yu et al., 2023). The inflammatory response in the ovary could indicate an active anti-apoptotic signaling in some cell types or a response to dying cells. In addition, GSEA analysis revealed an enrichment of pathways associated with apical junctions. This could imply alterations in cell-to-cell signaling during DDR or a reorganization of cell junctions because of cell death. Among the RRGs, eight genes encode proteins associated with cell-cell junctions (*Trp73, Nectin4, Fermt1, Fermt3, Pkp3, Tjp3, Nox1, Hmcn2*) and 23 associated with the plasma membrane (*Plch2, Ifitm10, Ano3, Eda2r, Gpr132, Islr2, Scn4b, Slc6a3, Nectin4, Crhr1, Gramd2a, Pkp3, Tjp3, Fermt1, Ak1, Baiap3, Nox1, Itgb7, C8a, Hmcn2, Ms4a10, Dcxr, Nlrp6*). The implications of these changes at cell-cell junctions will require further investigation as they may indicate coordinated signaling between pregranulosa cells and oocytes within the follicle or extrinsic signaling from other cell types in the ovary.

### 2.4. Ovarian radiation-response genes are enriched for p53 and p63 targets

**Fig. 4.**
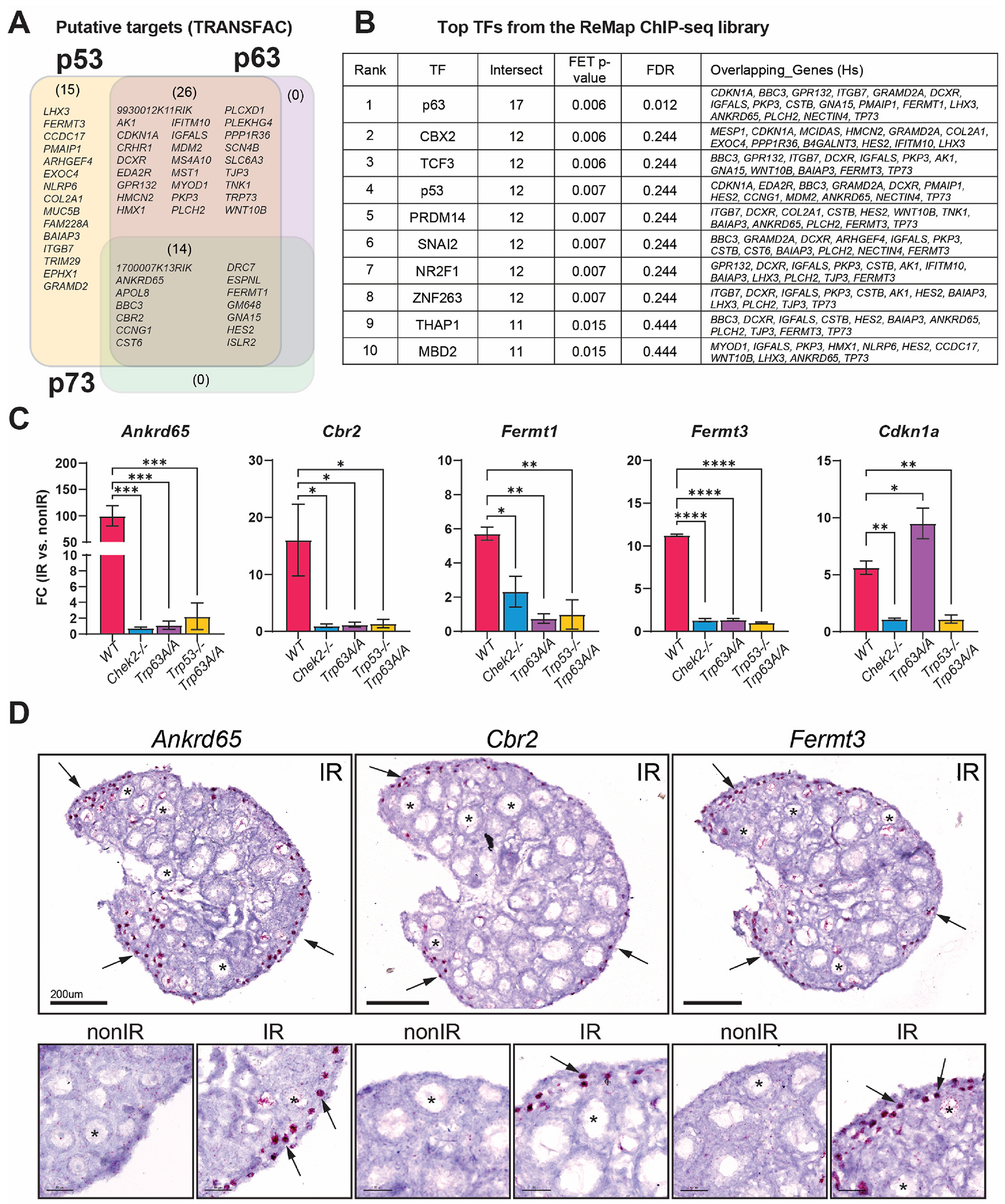
Radiation induces expression of TAp63 and p53 target genes. **A**) Venn diagram showing putative targets of p53, p63, and p73 transcription factors (TF) among RRGs based on TRANSFAC database. **B**) Top TFs predicted to regulate RRG expression based on validated ReMap ChIP-seq datasets. **C**) RT-qPCR analysis of RRGs expression in ovaries from mutant mice lacking active CHEK2, TAp63, and p53. One-week-old females were exposed to 0.5Gy IR and ovaries were collected 6 h after radiation for gene expression analysis. **D**) RNAscope validation of oocyte-specific upregulation of novel RRGs in the ovary 6 h after IR. The red signal indicates the presence of the transcript. Arrows indicate oocytes.

In response to DNA damage, the CHEK2 kinase activates several effector proteins, including two transcription factors: p53 and p63 (Zannini et al., 2014). These factors are known to induce apoptosis (Dietz et al., 2002; Flores et al., 2002; Haupt et al., 2003; Hirao et al., 2000; Pietsch et al., 2008). We conducted a gene enrichment analysis using the g:Profiler TRANSFAC database to determine whether RRGs identified in the ovary are regulated by p53, p63, or other transcription factors (TFs). Our analysis revealed that ovary RRGs were enriched for putative p53 binding motifs (55 target genes), as well as for p63 (40), and p73 (14). However, these largely overlapped with p53 target genes (Fig. 4A). These findings suggest that CHEK2-dependent response to radiation in the ovary is predominantly mediated by p53 and TAp63. Interestingly, expression of *Trp73*, the third member of the family, was induced in the ovary after radiation in a CHEK2-dependent manner (Fig. 2C–E). To identify p53 and TAp63 gene targets, experimentally validated by Chromatin Immunoprecipitation (ChIP) and sequencing experiments (ChIP-seq), we performed ChIP-X Enrichment Analysis (ChEA) (Keenan et al., 2019). We used the ChEA3 database, which aggregates previously annotated TF targets from multiple ChIP-seq experiments, including the ReMap dataset that includes mouse, human, and fly tissues (Hammal et al., 2022). This approach identified p63 as the top-ranking TF and p53 as the fourth (Fig. 4B, C). The top 10 TFs associated with ovary-RRGs include other TFs previously linked to the DNA damage response, such as CBX (Baumann et al., 2020; Ginjala et al., 2011; Vissers et al., 2012), TCF3 (Andrysik et al., 2013; Giono et al., 2016), SNAI2 (Gross et al., 2019; Pérez-Caro et al., 2008) and THAP1 (Roussigne et al., 2003; Shinoda et al., 2021). Interestingly, novel ovary-RRGs such as *Cbr2, Ankrd65, Fermt1*, and *Fermt3*, were all confirmed to be regulated by either p53 or p63 (Fig. 4A, B). As the ReMap dataset does not include ovarian tissue, we subsequently investigated whether these novel ovary-RRGs are activated by p53, TAp63, or both. We conducted RT-qPCR analysis on non-irradiated and irradiated ovaries collected 6 h post-treatment from females lacking active TAp63 (*Trp6*3*^A/A^*) (Emori et al., 2023) or p53 (Jacks et al., 1994). Due to the increased embryonic and early postnatal lethality of *Trp53*^*−/−*^ females, we used double mutants (*Trp6*3^*A/A*^; *Trp53*^*−/−*^) to assess the role of p53 in the ovary (Armstrong et al., 1995). Interestingly, females lacking both active TAp63 and p53 showed improved survival. Wild-type and *Chek2*^*−/−*^ ovaries were used as controls. RT-qPCR confirmed upregulation of *Cbr2, Ankrd65, Fermt1, Fermt3*, and *Cdkn1a* in wild-type but not in *Chek2*^*−/−*^ ovaries corroborating the bulk RNA-seq results. Furthermore, upregulation of *Cbr2, Ankrd65, Fermt1*, and *Fermt3* was abolished in ovaries lacking active TAp63, suggesting that these four genes are regulated by TAp63, not p53, in the ovary. Conversely, upregulation of *Cdkn1a*, a known p53-specific target downstream of CHEK2, was still observed in ovaries deficient for TAp63 but not in ovaries lacking p53 or CHEK2. This confirms that *Cdkn1a* is exclusively induced by p53, not TAp63, in the ovary. These findings confirmed the CHEK2-dependent upregulation of these ovary-RRGs and demonstrated that *Cbr2, Ankrd65, Fermt1*, and *Fermt3* are induced by TAp63 in the ovary (Fig. 4C). As TAp63 is exclusively expressed in oocytes, we predicted that these genes will be upregulated in oocytes. To test this, we performed RNA *in situ* hybridization (RNA-ISH) using the RNAscope system in non-irradiated and irradiated ovaries collected 6 h after IR. We observed a strong signal for all three genes near the periphery of the ovary, where PFs reside, exclusively in irradiated oocytes (Fig. 4D). Our results indicate that CHEK2 mediates the response to radiation-induced damage mainly via TAp63 in oocytes but also suggests a role for p53 in ovarian DDR.

### 2.5. Radiation elicits a stronger response in oocytes compared to somatic cells

**Fig. 5.**
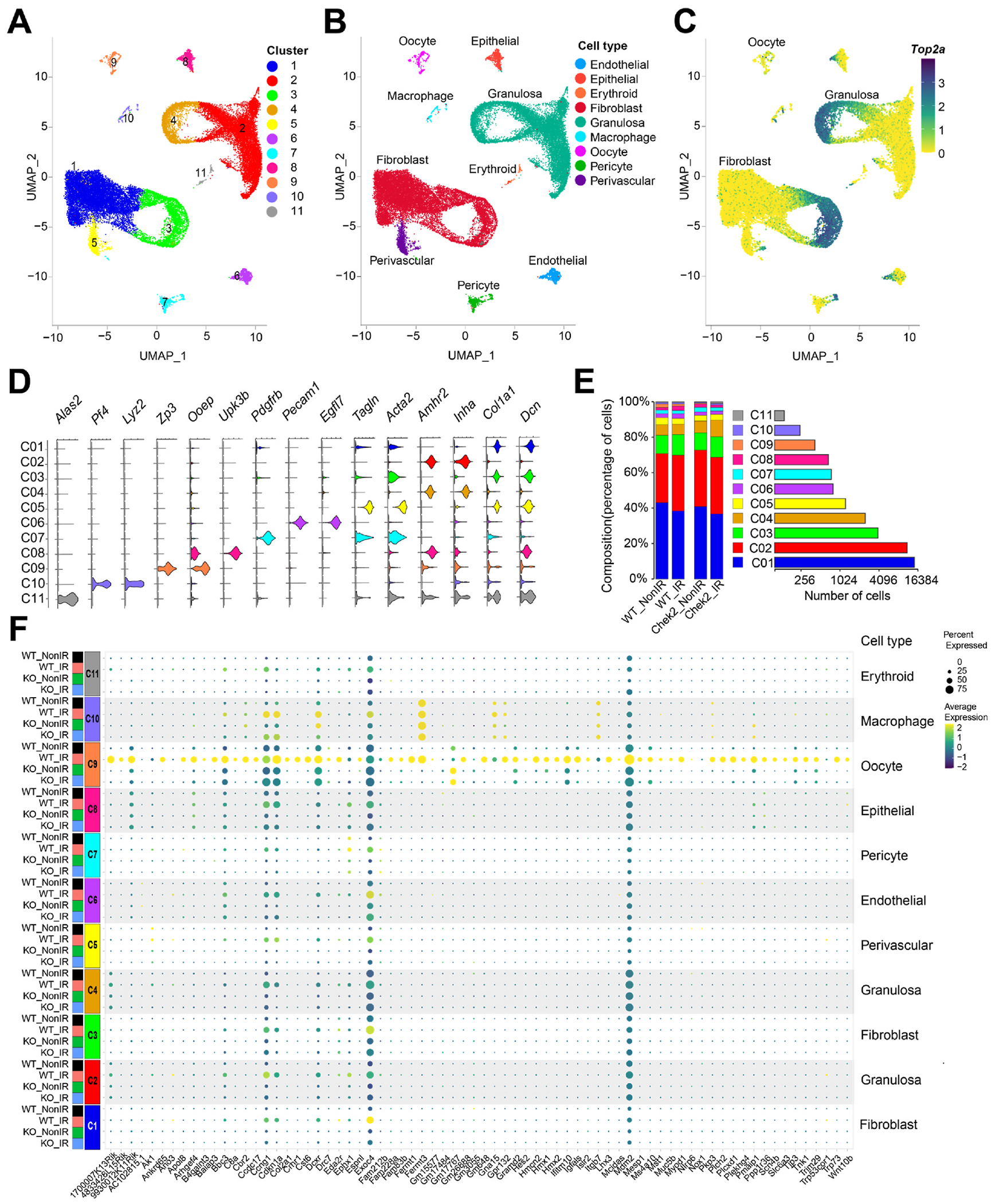
Radiation Responsive Genes are predominantly induced in oocytes. UMAP plots of all cells from wild-type and *Chek2*^*−/−*^ ovaries with and without radiation identified 11 clusters (**A**) representing nine cell types (**B**). **C**) UMAP plot showing expression of proliferation marker *Top2a*. **D**) Clusters were identified by the expression of cell-type-specific markers shown as violin plots. **E**) Distribution of cells among clusters. **F**) Bubble plot comparing expression of ovary-RRGs from bulk analysis across individual clusters and cell types. Bubble size is proportional to the percentage of cells in a cluster expressing a gene, and color intensity is proportional to average scaled gene expression within a cluster.

**Fig. 6.**
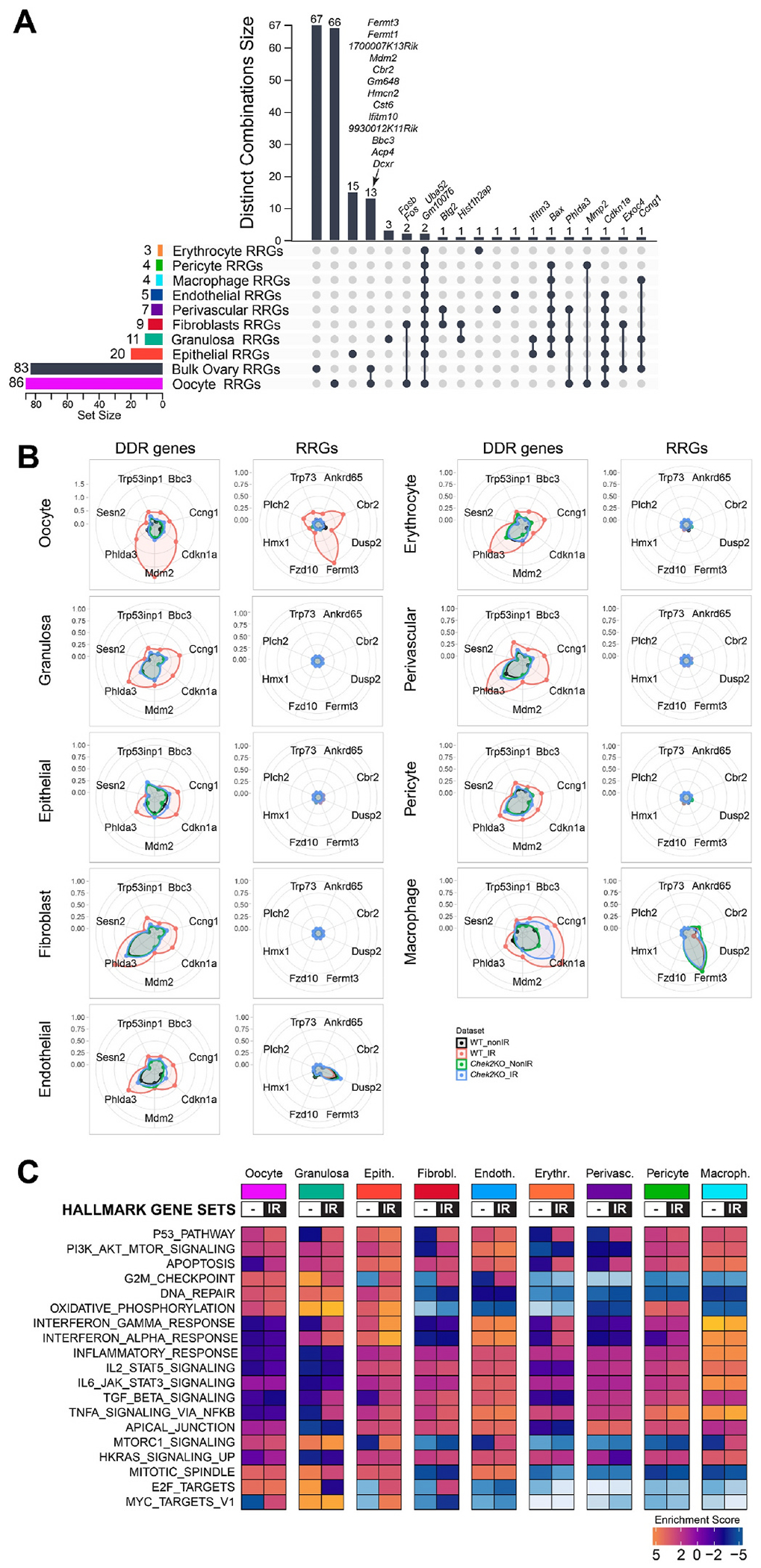
Radiation response varies between different cell types in the ovary. **A**) UpSet plot showing RRGs specific to a cell type and RRGs shared between cell types (single filled circle and filled circles connected with vertical lines, respectively). The vertical bar plot indicates unique or overlapping RRGs in clusters. The horizontal bar plot indicates the number of RRGs per cell type. **B**) Radar charts comparing upregulation of known DDR genes and RRGs in different cell types in wild-type and *Chek2*^*−/−*^ ovaries. Concentric rings represent log2FC. **C**) Heatmap showing different hallmark gene sets activated in specific cell types after radiation.

The majority of ovary-RRGs identified by bulk RNA-seq are expressed in various cell types and tissues, with some never reported to be expressed in untreated ovaries (Fig. S1). This raises the question of whether ovary-RRGs are induced in a specific cell type or if bulk RNA-seq can detect responses in primordial oocytes. Although CHEK2 is expressed in all cell types in the ovary, primordial oocytes are the most sensitive to IR and undergo apoptosis within 24 h post-IR (Stringer et al., 2020). This contrasts with most somatic cells in the ovary, including pregranulosa cells, which survive and persist longer, even in the absence of oocytes (Fig. S3). To dissect how CHEK2 regulates cell type-specific responses to IR-induced damage, we conducted single-cell RNA sequencing (scRNA-seq) in wild-type and *Chek2*^*−/−*^ ovaries exposed to the same IR regimen as in bulk experiments (6 h post-IR, 0.5 Gy vs. sham). We identified clusters corresponding to different cell types based on well-established markers, including *Dppa3, Sycp3, Dazl, Zp3, Gdf9* for oocytes, and *Inha, Inhbb, Amh, Amhr2* for granulosa cells (Fig. 5A–D and Fig. S2A). Overall, 11 clusters were identified, representing 9 cell types: oocytes, granulosa, fibroblasts, endothelial, epithelial, erythroid, macrophages, pericytes, and perivascular cells (Fig. 5B). Clusters 3 (fibroblasts) and 4 (granulosa cells) represent actively dividing cells based on high expression of a proliferation marker *Top2a* (Fig. 5C). Oocytes represented 1–2% of all cells in the ovary, compared to approximately 38% of granulosa cells and 50% of fibroblasts (Fig. 5E). To determine whether ovary-RRGs from bulk analysis were induced in oocytes or other cell types, we analyzed their expression in individual clusters. We found that the majority of ovary-RRGs were predominantly induced in irradiated oocytes (cluster 9) (Fig. 5F). Moreover, their induction was not observed in *Chek2*^*−/−*^ cells. Next, we investigated IR-induced changes in gene expression across clusters and cell types. We found that the oocytes have the highest number of differentially expressed RRGs compared to other cell types (Fig. 6A and Fig. S2B–D). 86 genes were differentially expressed at FC ≥ 1.5 and FDR ≤ 0.05 in the oocyte cluster. Among the genes were *Cbr2, Fermt1*, and *Fermt3*, which were previously identified by bulk analysis. The second highest upregulation of gene expression was observed in ovarian surface epithelium (20 genes) (Fig. 6A). Epithelial cells showed strong signature of interferon-induced immune response (*Isg15, Rsad2, Stat1, Tnfrsf12a*) and interferon-inducible genes (*Ifit1, Ifit3, Ifitm3, Iigp1, Irgm1*) (Der et al., 1998; Samarajiwa et al., 2009) (Supplementary Data 2). Granulosa cells, the second most abundant cell type in the ovary, displayed changes in the expression of 11 genes, including *Cdkn1a* and *Ccng1*, indicating induction of cell cycle arrest. As in bulk RNA-seq, very few DEGs were identified in *Chek2*^*−/−*^ cell clusters (Supplementary Data 2). These results indicate that IR-induced damage in the ovary elicits the strongest response in oocytes. This is supported by cellular changes observed in the ovary after IR, such as a high level of DNA damage marker γ-H2AX in oocytes compared to somatic cells (Fig. 1B) and persistence of pregranulosa cells after the loss of primordial oocytes (Fig. S3). Next, we compared the overlap between ovary-RRGs from bulk analysis and RRGs from each ovarian cell type (FC ≥ 1.5; FDR ≤ 0.05). The largest overlap was with the RRGs in the oocyte cluster (oocyte-RRGs) and included *Cbr2, Dcxr, Fermt1, Fermt3, Ifitm10, Mdm2, Cdkn1a, Gm648, Hmcn2, Bbc3, 9930012K11Rik, Cst6*, and *1700007K13Rik* (Fig. 6A). *Uba52, Cdkn1a,* and *Bax*—known DDR, cell cycle arrest, and apoptosis factors—were differentially expressed in multiple cell types. To assess DDR response patterns in gene expression across different cell types, we visualized the expression of core DDR genes and selected RRGs from this study using radar charts (Fig. 6B). Known DDR genes such as *Cdkn1a, Bbc3 Ccng1*, and *Mdm2* were induced in multiple cell types, while *Cbr2, Fermt1, Fermt3, Ankrd65, Trp73*, and a few other RRGs were exclusively induced in oocytes (Fig. 6B and Fig. S4). Interestingly, *Mdm2*—a negative regulator of p53—showed the highest induction and expression in irradiated oocytes compared to other cell types. This may suggest a unique mechanism limiting p53 activity in primordial oocytes during DDR (Fig. 6B). Among oocyte-RRGs that were not identified by the bulk analysis were *Perp, Ddit4, Fos*, and *Phlda3*, which have all been previously implicated in DDR and p53 pathway (Attardi et al., 2000; Ellisen et al., 2002; Kawase et al., 2009). To uncover the differences in how individual cell types respond to IR-induced damage, we conducted GSEA utilizing hallmark gene sets and pathways (Fig. 6C). Given the lack of significant response in *Chek2*^*−/−*^ ovaries, our analysis focused on wild-type ovaries. We observed clear differences in pathway activation between cell types. The strongest activation of apoptosis was detected in oocytes, erythroid, and perivascular cells. G2M checkpoint activation was detected in fibroblasts, epithelial, and endothelial cells. The p53 pathway was activated in oocytes, granulosa cells, fibroblasts, erythroid, and perivascular cells. Oocytes and epithelial cells showed induction of the c-MYC signaling, which is involved in p53-induced apoptosis (Phesse et al., 2014). The INF-α, INF-γ, and TNFα signaling via NF-κB— previously identified in bulk RNA-seq analysis—were activated in granulosa and epithelial cells. Previous studies reported elevated levels and overrepresentation of DNA repair genes in oocytes (Pan et al., 2005; Stringer et al., 2018). This suggests that oocytes are capable of efficient DNA repair. To test whether CHEK2 deficiency impairs DNA repair machinery, we examined constitutive expression profiles of 160 genes involved in DNA DSB repair in non-irradiated wild-type and *Chek2*^*−/−*^ cells (Ciccia and Elledge, 2010; Milacic et al., 2024; Rothkamm et al., 2003). In agreement with previous reports, oocytes showed elevated levels of most DSB repair genes compared to other cell types (Fig. S7A). Additionally, increased expression of many repair genes was also observed in mitotically active granulosa and fibroblast clusters (3 and 4). Further comparison revealed similar expression profiles of DNA repair genes in oocytes and granulosa cells in wild-type and *Chek2*^*−/−*^ ovaries (Fig. S7B). This suggests that oocyte’s capacity to repair damaged DNA is likely not affected by CHEK2 deficiency, at least at the transcriptional levels, although differences may arise at the level of protein activity. In summary, transcriptional profiling reveals that IR-induced damage is more harmful to primordial oocytes and results in a stronger response compared to other cell types, despite the expression of many DNA repair genes. Moreover, analysis at the single-cell level reveals that IR-induced damage activates different response pathways in different cell types.

### 2.6. Radiation induces unique cellular responses in oocytes

**Fig. 7.**
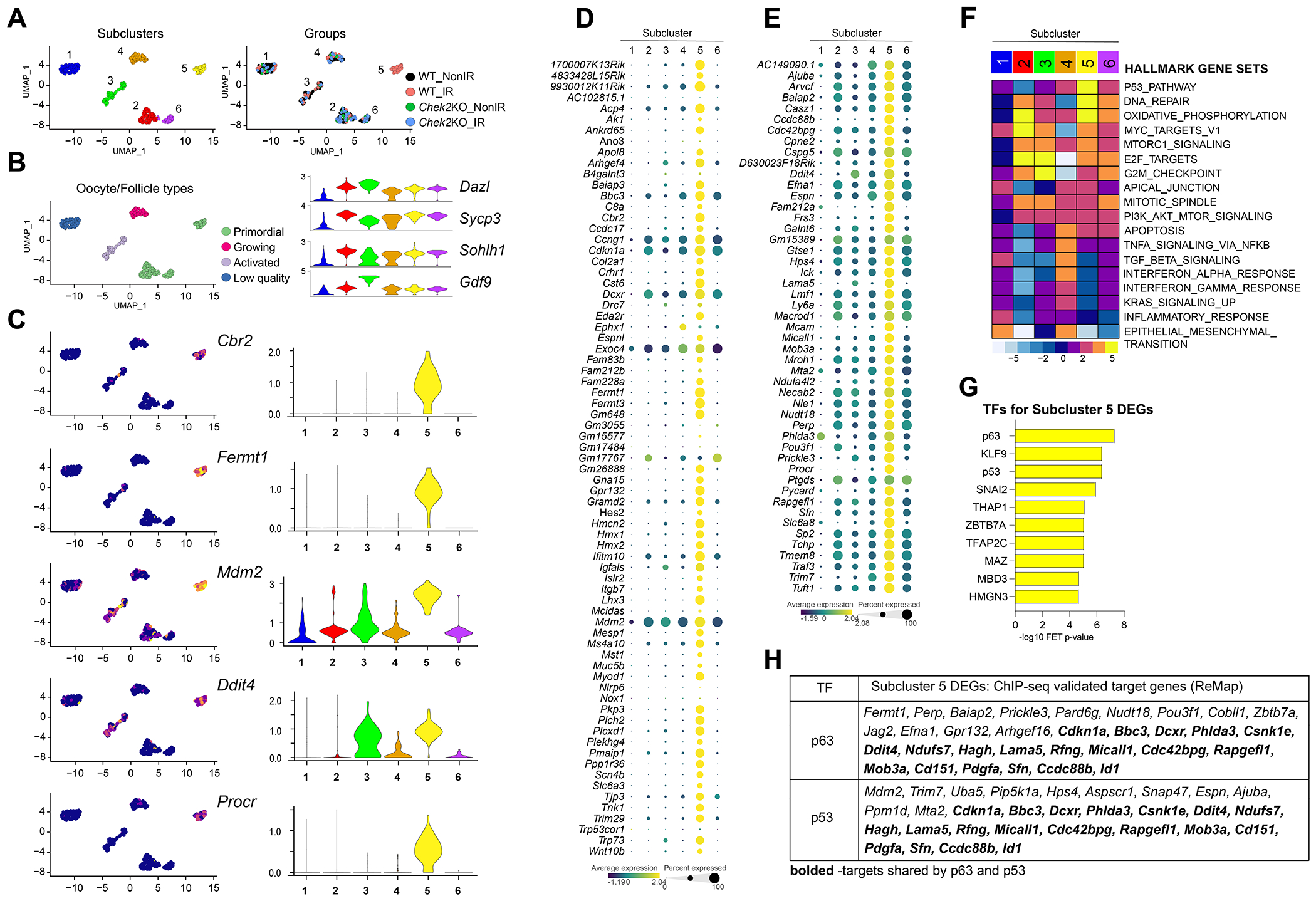
Oocytes display unique and diverse transcriptional changes in response to radiation. **A**) UMAP plots of oocyte subclusters identified by *de novo* reclustering. Subcluster 5 is comprised of solely wild-type irradiated oocytes. **B**) Markers of oocyte development shown as violin plots used to identify corresponding follicle stages. **C**) Gene expression levels of representative oocyte-RRGs overlaid on UMAP plots and shown by violin plots. **D**) Bubble plot comparing expression of ovary-RRGs from bulk analysis across six oocyte subclusters. **E**) Bubble plot showing subcluster-specific expression of additional oocyte-RRGs identified by subcluster analysis. **F**) Heatmap showing subcluster-specific gene set enrichment for different Hallmark gene sets. **G**) TF enrichment analysis for subcluster 5 RRGs using ChEA3 ReMap dataset. **H**) List of RRGs in subcluster 5 previously validated as p63 and p53 targets.

To enhance our understanding of the oocyte-specific response to IR-induced damage, we re-clustered both irradiated and non-irradiated oocytes from wild-type and *Chek2*^*−/−*^ females into six distinct subclusters. Interestingly, we identified a unique group, subcluster 5, consisting exclusively of irradiated oocytes from the wild-type group, while the remaining five subclusters included oocytes from all four groups (Fig. 7A). 44% of all irradiated wild-type oocytes were in subcluster 5, suggesting that the remaining irradiated oocytes in other subclusters may be at an earlier stage of DDR or from different follicle types that are resistant to radiation-induced DNA damage. Subclusters 2, 5, and 6 exhibited a transcriptional signature of primordial oocytes characterized by higher expression of genes related to meiosis (*Sycp1, Sycp3*), early oocyte development (*Solhl1*), and low expression of genes associated with oocyte growth (*Gdf9* and *Zp1/2*) (Figs. 7B and S5). In contrast, cells in subcluster 3 expressed high levels of *Gdf9* and *Zp1/2* indicative of growing oocytes in primary and secondary follicles (Figs. 7B and S5). Subcluster 4 likely represents activated oocytes in primordial or transitional follicles due to the downregulation of *Sycp1, Sycp3*, and *Solhl1*, as well as low expression of *Gdf9* and *Zp1/2* (Figs. 7B and S5). Subcluster 1 most likely contains low-quality cells based on low expression of oocyte marker genes and overall low gene count (Figs. 7B and S5). Subcluster 5 was distinguishable for the differential expression of 147 genes (FC ≥ 1.5; FDR ≤ 0.05) (Supplementary Data 3). Among them, 15 genes previously identified as ovary-RRGs in bulk ovary analysis, *Fermt1, Fermt3, Cbr2, 1700007K13Rik, Mdm2, 9930012K11Rik, Cdkn1a, Cst6, Ifitm10, Acp4, Dcxr, Hmcn2, Bbc3, Gpr132*, and *Gm648* (Fig. 7C, Fig. S5). We further examined the expression of all ovary-RRGs from the bulk analysis across the oocyte subclusters and observed that the majority were specifically upregulated in subcluster 5 (Fig. 7D). The fact that subcluster 5 oocytes represent 44% of all oocytes in the wild-type irradiated sample indicates that they largely contributed to the RRG signature in the ovary. However, many ovary-RRGs did not reach statistical significance in single-cell and subcluster analysis, potentially due to oocyte-to-oocyte variability in expression levels. The analysis of subcluster 5 identified additional oocyte-RRGs. Some are linked to p53 and apoptosis such as *Ddit4* (Ellisen et al., 2002), *Perp* (Ihrie et al., 2003) and *Pycard* (Ohtsuka et al., 2004), and others function as cell surface receptors such as *Lama5* (Spenlé et al., 2013), and *Mcam* (Wang and Yan, 2013) (Fig. 7C, E and Supplementary Data 3). RRGs from both bulk and subcluster analyses demonstrated CHEK2-dependent upregulation in the oocyte cluster (Fig. S6). GSEA revealed a strong enrichment for pathways related to p53, DNA repair, and oxidative phosphorylation in subcluster 5 oocytes (Fig. 7F). Enrichment for mTORC1, E2F, MYC targets, apical junctions, G2M checkpoint, and apoptosis was also observed in subcluster 5. While p53 and apoptotic signaling were expected in response to IR, the upregulation of pathways related to cell junctions detected by both bulk and single-cell approaches is noteworthy. Cell-to-cell communication through gap junctions can propagate an IR-damage response between cells via bystander effect, and the desmosomal protein PERP—upregulated in oocytes after IR (Fig. 7E)—is a known apoptotic factor (Attardi et al., 2000; Tang et al., 2023). To expand on our findings from the bulk analysis, we performed TF enrichment analysis for subcluster 5 RRGs using ChIP-seq data collected from the ReMap database, and again identified p63 as the top-ranking TF, followed by KLF9 and p53 (Fig. 7G). Two other transcriptional regulators already identified in bulk ovary analysis (Fig. 4B), SNAI2 and THAP1, were ranked higher for oocyte-RRGs (Fig. 7G). Interestingly, p63 and THAP1 are predominantly expressed in oocytes compared to other ovarian cells (Fig. S8), suggesting they may propagate DDR signaling specific to oocytes. p53 and KLF9 are expressed in all cell types while SNAI2 is highly expressed in fibroblasts (Fig. S8). Overall, 32 DEGs in subcluster 5 were previously experimentally validated as targets for p63, 30 for p53, and 19 shared by both factors (Fig. 7H). In summary, *de novo* re-clustering of oocytes from control and irradiated wild-type and *Chek2*^*−/−*^ ovaries identified primordial oocytes undergoing active DDR. These oocytes show strong activation of the p53 pathway, DNA repair, and oxidative phosphorylation. Interestingly, activation of the apical junction pathway and upregulation of plasma membrane/cell surface proteins in primordial oocytes in subcluster 5 suggests an intriguing possibility of coordinated DDR signaling at the interface between primordial oocytes and pregranulosa cells. Moreover, these results indicate that, although TAp63 primarily mediates DDR and apoptosis in oocytes, the strong signature of p53-related signaling suggests an active role for p53 in the oocyte’s response to DNA damage. Further studies are needed to determine which DDR processes in oocytes are regulated by p53, and to which extent DDR in pregranulosa cells affects oocyte survival or death.

### 2.7. Radiation induces a moderate response in pregranulosa cells in primordial follicles

**Fig. 8.**
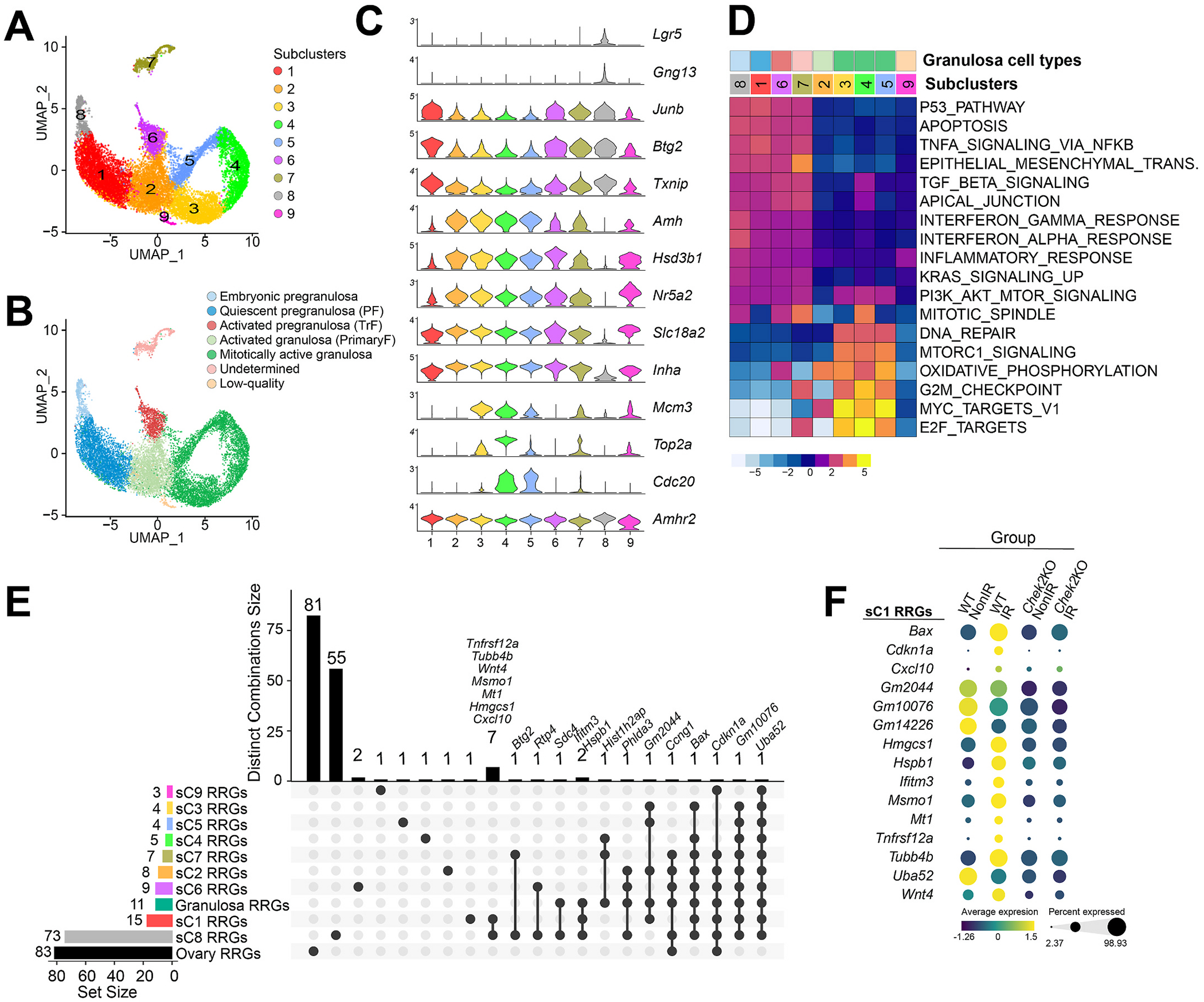
Pregranulosa in primordial and granulosa cells in growing follicles differ in pathway activity and response to radiation. UMAP plots of granulosa cells subclusters (**A**) and associated follicle stage (**B**) identified by expression of genes representing follicle development shown as violin plots (**C**). PF - primordial follicle, TrF - transitional follicle, PrimaryF - primary follicle. **D**) Heatmap showing granulosa cell subcluster and follicle type-specific gene set enrichment for different Hallmark gene sets using GSEA. **E**) UpSet plot showing RRGs specific and shared between granulosa cell subclusters (sC1–sC9) (single filled circle and filled circles connected with vertical lines, respectively). Vertical bar plot indicates unique or overlapping RRGs in subclusters. The horizontal bar plot indicates the number of RRGs per cluster. **F**) Bubble plot comparing expression of RRGs from pregranulosa cells of primordial follicles (subcluster 1) in wild-type and *Chek2*^*−/−*^ cells.

Radiation-induced damage eliminates oocytes in primordial follicles but not in growing primary and secondary follicles (Fig. 1A). Therefore, we hypothesized that the radiation response differs between pregranulosa cells from primordial follicles and granulosa cells from growing follicles. To identify pregranulosa cells, we *de novo* re-clustered granulosa clusters 2 and 4, which resulted in nine distinct subclusters (Fig. 8A and Fig. S9A). We classified cells in subcluster 1 as pregranulosa in primordial follicles based on the expression of previously described quiescence genes such as *Junb, Btg2*, and *Txnip* (Meinsohn et al., 2021) and lack of follicle activation markers *Amh, Hsd3b1, Nr5a2, Slc18a3* and *Fam13a* (Meinsohn et al., 2021; Niu and Spradling, 2020) (Fig. 8B, C). Subcluster 2 showed downregulation of quiescence markers and upregulation of follicle activation markers such as *Amh, Inha*, and *Hsd3b1*; therefore, they were classified as granulosa cells in activated primary follicles. Subclusters 3–5 contained mitotically active granulosa cells from growing primary follicles characterized by signatures of follicle activation (*Amh* and *Hsd3b1*) and proliferation (*Mcm2–7*, *Top2a, and Cdc20*). Subcluster 6 expressed high levels of *Kitl* and *Hsd3b1* as well as quiescence markers *Junb* and *Btg2*, suggesting that they were pregranulosa cells that have initiated follicle activation and may belong to the transitional follicle (Fig. 8B, C). Cells in subcluster 8 expressed epithelial markers *Gng13 and Lgr5* along quiescence genes, possibly representing the remnant population of embryonic epithelial pregranulosa cells (Meinsohn et al., 2021; Niu and Spradling, 2020). The identity of cells in subcluster 7 remains undetermined as they expressed granulosa and fibroblast-related genes (Fig. 8B, C). Cells in subcluster 9 were classified as low-quality due to low gene counts. In contrast to oocytes, irradiated and non-irradiated cells from wild-type and *Chek2*^*−/−*^ ovaries were distributed across all subclusters (Fig. S9B). To evaluate whether pregranulosa or other granulosa subtypes contributed to the radiation response signature in the ovary, we analyzed the expression of ovary-RRGs. Unlike for oocytes, there was no specific granulosa subcluster with a strong radiation-response signature, and most ovary-RRGs were not expressed in granulosa cells with the exception of known DDR genes such as *Bbc3, Ccng1*, and *Mdm2* (Fig. S9C). Interestingly, GSEA revealed differences in pathway activity between pregranulosa cells in subclusters 1, 6, and 8 and granulosa cells in growing follicles in subclusters 2–5 (Fig. 8D). These differences may be linked to varying radiation responses within these two types of follicles as individual units. Pregranulosa cells showed a stronger signature for the p53 pathway, apoptosis, apical junction, TNFα signaling via NF-κB, and interferon signaling while granulosa cells in growing follicles showed a stronger signature for the DNA repair, MTORC1 signaling, oxidative phosphorylation, and G2M checkpoint (Fig. 8D). Although the identity of subcluster 7 cells remains unclear they showed a signature more similar to pregranulosa cells (Fig. 8D). Differential gene expression analysis in individual subclusters revealed that pregranulosa cells in primordial follicles (subcluster 1) exhibited a moderate response to radiation with fifteen genes showing significant changes in expression in wild-type ovaries after radiation (FC ≥ 1.5; FDR ≤ 0.05), including *Cdkn1a, Bax, Hspb1, Hmgcs1, Msmo1, Tnfrsf12a, Tubb4b, Ifitm3*, and *Cxcl10* (Fig. 8E and Supplementary Data 4). Interestingly, embryonic epithelial pregranulosa cells in subcluster 8 exhibited the strongest response to radiation, with 73 differentially expressed genes (FC ≥ 1.5; FDR ≤ 0.05) including *Cxcl10, Isg15, Ifitm3, Ifit1, Hmox1, Mt1, Tnfrsf12a, Hsp90aa1, Hspb1, Cdkn1a* (Fig. 8E). Other subclusters showed significant differential expression of only a small number of genes in wild-type irradiated cells (FC ≥ 1.5; FDR ≤ 0.05), including *Cdkn1a, Uba52*, and *Bax* (Fig. 8E, F and Supplementary Data 4), in agreement with findings from the whole granulosa cell cluster analysis. In summary, analysis of the radiation response across different types of granulosa cells revealed a moderate transcriptional response in pregranulosa cells from radiation-sensitive primordial follicles and a weak response in granulosa cells from radiation-resistant growing follicles.

## 3. Discussion

Females are born with a finite supply of immature PFs, which are expected to last throughout their reproductive lifespan until menopause. As we increasingly recognize that various exogenous and endogenous sources can cause DNA damage in primordial oocytes enclosed in PF, leading to their accelerated depletion, it becomes critical to identify the factors that regulate the oocyte’s response to DNA damage and its survival. This is important because the loss of immature follicles results in loss of fertility and hormonal dysregulation in females. In this study, we employed both bulk and single-cell transcriptomics to uncover the molecular mechanisms of DDR in the ovary that led to the elimination of primordial follicles in response to DNA damage caused by ionizing radiation. Our approach revealed that the response to DNA damage induced in ovaries, by a relatively low but oocyte lethal dose of radiation, is solely dependent on CHEK2 downstream signaling as the transcriptional response was almost entirely abolished in its absence. Radiation-induced DNA damage disproportionately affected oocytes and induced the strongest response at the transcriptional level driven by two CHEK2 targets: TAp63 and p53. We further demonstrate that the ovarian response to radiation involves the activation of interferon and inflammatory pathways in the ovarian soma and signaling at the cell-cell junctions likely between the oocyte and pregranulosa cells. Our findings reveal novel genes and unique responses in oocytes DDR, which may help explain their sensitivity to various genotoxic insults.

The single-cell composition of one-week-old ovaries, determined through transcriptional cell clustering, reveals the presence of nine major cell types. Among these, fibroblasts (or fibroblast-like stromal cells) and granulosa cells are the most abundant, together accounting for nearly 90% of all cells. Oocytes constitute around 2%, while the remaining cell types include endothelial, epithelial, erythroid, macrophages, pericytes, and perivascular cells. Following IR, oocytes exhibit the highest number of differentially expressed genes. This suggests that primordial oocytes are exquisitely sensitive to DNA damage and harbor a unique DDR. Somatic cells in the G2/M phase of the cell cycle are most sensitive to DNA damage due to the limited time for repair before chromosome segregation and subsequent cell division. Therefore, it may not be surprising that primordial oocytes, which are suspended in the dictyate stage of meiotic prophase I, similar to the G2 phase, are highly sensitive to DNA damage. However, despite remaining arrested for many months or years—which would give them enough time to repair the damage before resuming meiosis—they seem to default to apoptosis rather than repair despite elevated expression of key DNA repair genes.

The examination of changes in gene expression indicates that oocytes exhibit the strongest response to DNA damage in the ovary. Radiation treatment led to upregulation of multiple known DDR and apoptotic genes in oocytes including *Cdkn1a, Bbc3, Ddit4, Perp*, and *Phlda3*. This is in agreement with a previous study in purified primordial oocytes which reported an enhanced expression of pro-apoptotic genes (Fester et al., 2022). However, we also identified other upregulated genes in irradiated oocytes that point to additional response pathways that were not previously implicated in the DDR in ovaries. Although the function of *Ankrd65* is unknown, Ankyrin repeats mediate protein-protein interactions, and many proteins with ankyrin repeats have been implicated in DNA damage response (Dhyani et al., 2015; Hall et al., 2016; Neilsen et al., 2008). This suggests that ANKRD65 plays a role in DDR and damage repair, specifically in oocytes. The upregulation of *Cbr2* and *Dcxr*—which belong to a family of carbonyl oxidoreductases—reveals a potential mechanism of detoxification in response to oxidative damage specifically in oocytes (Ebert et al., 2015). These proteins can reduce endogenous and exogenous carbonyl compounds, including those derived from lipid peroxidation of mitochondrial membranes, and can provide protection against reactive oxygen and reactive carbonyl species (ROS and RCS). CBR2 is predominantly expressed in the lungs and is reported to localize to mitochondria but its exact function remains unknown (Matsuura et al., 1994). *Cbr2*^*−/−*^ females seem to be fertile, as are *Chek2*^*−/−*^, therefore more in-depth studies are needed to determine the role of CBR2 in ovarian DDR and follicle survival (de Angelis et al., 2015). DCXR is a multifunctional protein involved in sugar metabolism, carbonyl detoxification, cell adhesion, and male fertility (Ebert et al., 2015). *C. elegans* ortholog of DCXR (DHS-21) plays a role in detoxification of carbonyl compounds, and *dhs-21* mutants have shorter lifespans presumably due to decreased defense against oxidative damage (Son et al., 2011). *Dcxr*-deficient mice die before wean age, thus the role of DXCR in ovaries and female fertility was not assessed (de Angelis et al., 2015). However, DCXR was detected in human oocytes by Human Protein Atlas (Uhlén et al., 2015). This suggests that CBR2 and DCXR may protect oocytes from oxidative damage induced by IR and endogenous sources, however they may not be required for oocyte survival in the absence of pro-apoptotic signaling. Although a *Cbr2* ortholog was not found in humans, it is possible that DCXR alone fulfills this function in human oocytes. Intriguingly, DCXR is also implicated in cell adhesion and has been shown to colocalize with E-cadherin at cell-cell junctions (Cho-Vega et al., 2007; Ebert et al., 2015). Importantly, *Dcxr* is not the only gene induced by IR in oocytes associated with cell-cell junctions, suggesting a potential role for communication between oocytes and pregranulosa cells in the regulation of oocyte survival. *Fermt1* and *Fermt3*, also known as kindlin-1 and 3, are best known for their role in cell-extracellular matrix adhesion and integrin activation (Larjava et al., 2008; Plow and Qin, 2019). Interestingly, FERMT1 plays a role in oxidative stress response, and its deficiency results in increased sensitivity to oxidative stress (Emmert et al., 2017). The exact mechanism by which integrin-mediated adhesion may regulate oocyte survival remains unknown. Nevertheless, it has been demonstrated that the activation of integrins and cell adhesion can regulate cell survival in response to DNA damage through the modulation of p53 and p73/c-Abl, thereby potentially linking kindlins and apoptosis in oocytes (Lewis et al., 2002; Truong et al., 2003). Furthermore, we found that IR causes an upregulation of the cell-adhesion gene *Perp* in oocytes. PERP, a desmosomal protein, is a known target of p53 and p63 and is involved in apoptosis (Awais et al., 2016; Ihrie et al., 2003; Roberts and Paraoan, 2020). The oocytes in PFs are surrounded by pregranulosa cells, which are similarly quiescent and non-proliferative. They support oocyte quiescence and survival through direct contact and bidirectional communication across cell-cell junctions (Eppig, 1991, 2018; Rodrigues et al., 2021). These findings imply that there may be a coordinated response to radiation between primordial oocytes and the surrounding pregranulosa cells.

The analysis of DDR in pregranulosa cells from primordial follicles revealed a moderate transcriptional response to radiation with fifteen differentially expressed genes. In addition to canonical DDR genes (*Cdkn1a* and *Bax*), pregranulosa cells upregulated other genes previously linked to radiation response. Two genes involved in the biosynthesis of cholesterol *(Hmgcs1* and *Msmo1*) were upregulated in pregranulosa cells after radiation. It has been reported that *Hmgcs1* regulates radiosensitivity of cancer cells by controlling cholesterol metabolism and mitochondrial gene expression (Zhang et al., 2023). Cholesterol is concentrated in the plasma membrane and thus may affect protein function at the interface between the oocyte and pregranulosa cells. Although there is evidence for the role of cholesterol in DDR, the underlying mechanisms remain unknown (Werner et al., 2019). Over expression of a heat shock protein HSPB1 (HSP25) has been shown to provide protection against radiation, therefore upregulation of *Hspb1* in pregranulosa cells could contribute to their radiation resistance (Baek et al., 2000; Park et al., 2000). Interestingly, the remnant population of embryonic pregranulosa cells showed the most robust response to radiation among granulosa cell types. It is unclear if these cells contribute to primordial follicles in the one-week-old ovary or persist in the ovary beyond puberty. In contrast, granulosa cells in growing follicles were largely unaffected by radiation at the transcriptional level, reflecting their tolerance to the radiation dose used in this study. The difference in oocyte’s response to radiation enclosed in primordial or growing follicles may be also linked to the differential activity of cellular pathways in the supporting granulosa cells within the follicle. Elevated activity of p53, apoptosis, apical junctions, interferon, and TNFα signaling pathways in pregranulosa cells may contribute to PF radiosensitivity. More studies are needed to better understand the communication between the oocyte and granulosa cells in the radiation-sensitive and radiation-resistant follicles.

Fibroblast cells in the ovarian stroma show an overall weak response to radiation. Upregulation of *Cdkn1a, Exoc4,* and *Btg2* in these cells indicates activation of DNA repair and pro-survival responses (Rouault et al., 1996; Torres et al., 2015). This implies that the IR dose used in this study, while lethal to primordial oocytes, is tolerated by fibroblasts. This is corroborated by previous studies where neither lower (0.1 Gy) nor higher (1 Gy) doses resulted in fibrosis which could arise after IR (Kimler et al., 2018; Quan et al., 2020). The ovarian surface epithelium showed the second highest number of differentially expressed genes (20 DEGs), exhibiting a strong interferon-induced immune response. This included genes such as *Isg15*, *Rsad2*, *Stat1*, *Tnfrsf12a*, and interferon-inducible genes *Ifit1*, *Ifit3*, *Ifitm3*, *Iigp1*, *Irgm1*. Multiple epithelial RRGs were also upregulated in pregranulosa cells (e.g., *Isg15*, *Ifitm3*, *Tnfrsf12a*), which may reflect their epithelial origin (Niu and Spradling, 2020). Most of our knowledge about the role of interferon signaling in response to IR-induced DNA damage comes from studies in cancers where it is often linked to cGAS/STING signaling (Deng et al., 2014; Goedegebuure et al., 2021; Storozynsky and Hitt, 2020; Wilkins et al., 2019). Interferon alpha was shown to induce apoptosis via the mitochondrial pathway and activation of BAX, which we see upregulated in epithelial cluster cells (Panaretakis et al., 2003). ISG15, a ubiquitin-like protein that conjugates to various proteins in response to interferon, was previously shown to be upregulated in response to DNA damage and has been implicated in the regulation of p53 and p63 (Sandy et al., 2020; Wardlaw and Petrini, 2023). Interestingly, while TAp63 is exclusively expressed in oocytes, p53 and CHEK2 are ubiquitously expressed in all cells within the ovary. Therefore, it is possible that extrinsic signaling from other cells in the ovary, including the neighboring pregranulosa and surface epithelium or fibroblasts in the stroma, may also play a role in regulating the fate of primordial oocytes either by direct cell-cell communication, or by stimulating changes in the microenvironment (Gaugler et al., 2007; Martinez-Zubiaurre and Hellevik, 2023; Widel et al., 2012). Intriguingly, a previous study reported a strong interferon response in purified oocytes after IR (Fester et al., 2022). Our study detected INF-α, INF-γ, and inflammatory signatures in ovaries by bulk RNA-seq but not in oocytes using scRNA-seq. It is possible that the protocol used for oocyte purification resulted in contamination with epithelial cells or incomplete separation of primordial oocytes from pregranulosa cells, which may be the actual source of the interferon signaling. Studies in cancer cells show that the upregulation of interferon-stimulated genes is associated with resistance to DNA-damaging radiotherapy (Padariya et al., 2021; Weichselbaum et al., 2008). This suggests that interferon response may contribute to the resistance of pregranulosa cells to radiation and their survival. Further studies are needed to determine the role of interferon signaling in primordial follicles and ovarian environment in response to DNA damage.

Although p53 is activated, and p53-regulated genes are upregulated in response to DNA damage in oocytes, much less is known about the role of p53 in primordial oocytes compared to TAp63. p53 is a multifunctional protein best known for regulating cell cycle arrest or inducing apoptosis in response to DNA damage (Wang et al., 2023). While most of our knowledge about p53 comes from studying cancer cells or other proliferating cells, it has also been implicated in regulating non-apoptotic programs such as metabolism, stemness, and neuronal differentiation (Lacroix et al., 2020; Olivos and Mayo, 2016; Tedeschi and Di Giovanni, 2009). Interestingly, a study in postmitotic cells in Drosophila revealed that p53 does not induce apoptosis after IR. Instead, it regulates metabolism, proteolysis, and DNA repair (Kurtz et al., 2019). It is possible that in quiescent meiotically arrested primordial oocytes, p53 is also not involved in triggering apoptosis; its primary role may be regulating oocyte survival. Moreover, it has been shown that cell type-specific and condition-dependent thresholds of p53 accumulation determine its pro-apoptotic activity (Hanson et al., 2019; Jiménez et al., 2022; Stewart-Ornstein et al., 2021). Our previous work has shown that alkylating agents induce more DNA damage in oocytes, leading to activation of p53-dependent apoptosis and primordial follicle loss (Emori et al., 2023). Therefore, the amount of cellular damage and specialized mechanisms likely regulate p53 activity in oocytes. Elevated expression of *Mdm2* in oocytes, which directs p53 degradation, suggests tight control of p53 activity in oocytes. Indeed, loss of MDM2 activity in oocytes causes premature depletion of PFs due to accumulation of p53 (Zhang et al., 2017). A better understanding of the DDR responses driven byTAp63 and p53—that may be beneficial to oocyte’s survival but are made ineffective by excessive (or precautious) TAp63-driven apoptosis—will be critical for elucidating the mechanisms of ovarian aging, and ovariotoxic effects of therapeutic or environmental genotoxic exposures.

## 4. Materials and methods

### 4.1. Animals

All procedures used in this study were approved by the IACUC at The Jackson Laboratory. C57BL/6J (#000664) and *Trp53*tm1Tyj/J (#002101) mice were obtained from The Jackson Laboratory. *Chek2*tm1b(EUCOMM)Hmgu mice were obtained from KOMP program at JAX. *Trp63*S621A mutant line was generated using CRISPR/Cas9 (Emori et al., 2023). For total body irradiation experiments, 7 to 9-day old females were irradiated using a Cesium-137 gamma irradiator. Females were exposed to sham or a single dose of 0.5 Gy administered at a rate of (~170 Rad/min). Ovaries were collected at 3 or 6 h after radiation for RNA or protein extraction or were fixed in 4% PFA for immunostaining. For whole ovary immunostaining and imaging, ovaries were collected one week after radiation following perfusion with PFA as previously described (Boateng et al., 2021).

### 4.2. Immunohistochemistry

Ovarian sections of 5 μm thickness were prepared and immunostained using standard procedures. Primary antibodies used in this study were mouse anti-p63 (4A4, Biocare Medical, CM163A), rabbit anti-pCHEK2(T68) (Bioworld Technology, BS4043), rabbit anti-phospho-p53(S15) (Cell Signaling, 9284), rabbit anti-DDX4 (Abcam, ab13840), mouse anti-γ-H2AX (Millipore, 05–636). The secondary antibodies used were Alexa Fluor (Invitrogen). Immunostaining for phospho-p53(S15) was performed with Starr Trek reagent (Biocare Medical, STUHRP700H). Imaging was performed using a Leica DM550 microscope and LAS X software (Leica).

### 4.3. Whole ovary immunostaining, optical clearing, and imaging

Immunostaining and optical clearing of whole ovaries was performed using CUBIC as described in (Boateng et al., 2021). The primary antibody incubation was carried out at room temperature with gentle rocking for 2–4 days. The primary antibodies used were rabbit anti-DDX4 (Abcam, ab13840) and secondary antibodies were Alexa Fluor (Invitrogen). Whole ovaries were imaged using the Leica DIVE/4Tune multiphoton and LAS X software (Leica). 3D rendering was prepared with IMARIS software (Bitplane).

### 4.4. In situ hybridization using RNAScope

Ovaries from 9-day-postpartum pups treated with sham or 0.5 Gy IR were fixed in 4% paraformaldehyde overnight at 4°C, embedded by freezing in O.C.T. media (Tissue-Tek), and sectioned into 10 μm thickness. Sections were processed using the Manual RNAscope 2.5 HD RNAscope kit (Advanced Cell Diagnostics (ACD) #322350) as described in the manufacturer’s instructions. In brief, 10-μm ovarian sections were pretreated with protease before hybridization with target probes: *Ankrd65* (ACD #843881), *Cbr2* (ACD #842871), and *Fermt3* (ACD #562571) and control probes: *Ppib* (ACD #313911), *DapB* (ACD #310043). Sections were incubated with amplifier probes AMP1 through AMP5, and then Fast-RED A/B solution was applied to sections for chromogenic staining of probes. Positive staining was identified as red, punctuate dots in the cells. Sections were counterstained with Hematoxylin Gills I and 0.02% ammonia water was used for blueing. Images were acquired using a bright field microscope (Leica DM5500) or NanoZoomer (C-13210-01). Experiments were performed with at least two ovaries per IR condition with a minimum of three replicates.

### 4.5. Quantitative RT-qPCR analysis

Ovaries were dissected from female pups exposed to sham or 0.5 Gy radiation 6 h after exposure (N = 3 per group). The ovaries were immediately frozen in liquid nitrogen and stored at −80°C until RNA extraction. Total RNA was extracted from pooled ovaries using a RNeasy Micro Kit (Qiagen, 74004) according to the manufacturer’s instructions, with an additional DNAse I treatment step to remove DNA contamination. A minimum of 500 ng of total RNA was reverse transcribed into cDNA using SuperScript IV reverse transcriptase (Invitrogen, 18091050) according to the manufacturer’s instructions. The cDNA samples were used as templates for quantitative real-time PCR (qPCR) using the ViiA7 Real Time PCR system (Life Technologies) and the Power Track SYBR Green PCR master mix (Applied Biosystems, A46109). The qPCR reactions were performed in triplicate. The primer sequences for the target genes and the reference gene *Gapdh* are shown in Table S4. The qPCR cycling conditions were as follows: 95°C for 2 min, followed by 40 cycles of 95°C for 15 s and 60°C for 1 min. The qPCR data were analyzed using the ViiA7 software, and the gene expression levels were calculated using the 2-ΔΔCT method, with GAPDH as the reference gene. The fold change in expression for each target gene between the sham and 0.5 Gy groups was calculated and plotted using PRISM 9.5.1 (GraphPad Software). Statistical analysis was performed using PRISM 9.5.1. One-way ANOVA with Bonferroni post hoc analysis was employed to determine differences between more than two groups. Values of P < 0.05 were considered statistically significant. Data are presented as means ± SEM.*P < 0.05; **P < 0.01; ***P < 0.001; P < 0.0001; n.s., non-significant.

### 4.6. Bulk RNA sequencing and analysis

Ovaries were dissected 6 h after sham or radiation treatment with 0.5 Gy and stored in RNAlater until extraction. RNA was extracted from paired ovaries (N = 6 per condition) using a miRNeasy micro extraction kit per the manufacturer’s instructions (Qiagen, 217084). RNA concentration and quality were assessed using the RNA Total RNA Nano Assay (Agilent Technologies). Libraries were constructed using the KAPA mRNA HyperPrep Kit (KAPA Biosystems). Library quality and concentration were checked using the D5000 Screen Tape (Agilent Technologies) and quantitative PCR (KAPA Biosystems). Barcoded libraries were then pooled and sequenced on the HiSeq2000 (Illumina) using TruSeq SBS Kit v4 reagents. The primary RNA-seq processing, quality control, and transcript-level quantitation were carried out using Nextflow-based pipeline nf-core/rnaseq (version1.4.3dev) (Ewels et al., 2020). Briefly, 100bp paired-end reads quality was checked using FastQC (v.0.11.9) (RRID:SCR_014583). Reads passing the quality thresholds were aligned and quantified using the Salmon quantification tool (RRID:SCR_017036) (Patro et al., 2017). Salmon v.1.3.0 was used to build an index and quantify transcript and gene expression against the reference transcriptome (Ensembl transcript release 105) using default parameters. Differential gene expression analysis was performed using the DESeq2 package (v.1.28.1) (RRID:SCR_015687) (Love et al., 2014). Differentially expressed genes were defined as significant with an adjusted p-value (FDR) < 0.05 and Fold change ≥ 2. The bulk RNA-seq results were visualized using heatmaps, volcano and box plots in Partek Genomics Suite (RRID:SCR_011860).

### 4.7. Single-cell RNA sequencing

7-day-old wild-type and *Chek2*^−/−^ females (N = 4 per group) were exposed to sham or 0.5 Gy radiation. Pooled ovaries were dissected 6 h after exposure and immediately dissociated into a single-cell suspension using the combined enzymatic-mechanical tissue dissociation protocol. In brief, ovaries were first incubated in collagenase IV (1 mg/ml, Worthington) and DNAse (0.02%, Worthington) solution in HBSS. After 15 min incubation at 37°C, trypsin was added to a final concentration of 0.125% and solution with ovaries was mixed by gentle pipetting. Following additional 10 min incubation at 37°C trypsin was inactivated by adding FBS and solution with ovaries was mixed by gentle pipetting with a wide-bore tip to mechanically facilitate the dissociation of the tissue into a single-cell suspension. Cell suspensions were filtered with a 40 μm mesh filter to remove debris and cell aggregates and spun down at 2000 rpm for 5 min in a swing-bucket centrifuge. Cell pellets were resuspended in 2% BSA PBS and single-cell suspensions were analyzed for viability and counted on a Countess II automated cell counter (Thermo Fisher). A total of 12,000 cells per sample were loaded onto a channel of 10X Chromium microfluidic chips for a targeted cell recovery of 6000 cells per lane. Single-cell capture, barcoding, and library preparation were performed according to manufacturer’s protocol (10x Genomics). Sample cDNA and library quality controls were performed using the Agilent 4200 TapeStation instrument and quantified by qPCR (Kapa Biosystems/Roche). Libraries were sequenced on a NovaSeq 6000 (Illumina) with the S2 100 cycle kit targeting 50,000 reads per cell.

### 4.8. Single-cell data processing and analysis

The raw sequencing reads from Illumina were aligned to the mouse reference genome mm10 using Cell Ranger V4 software (RRID:SCR_017344) with default parameters. Filtered gene counts generated after Cell Ranger were used for all the downstream analyses. The low-quality cells failing to meet the following threshold criteria were discarded, <1000 genes expressed or >20% mitochondrial transcripts or >50% ribosomal transcripts. In addition, the genes that were expressed in less than 3 cells were also discarded. The potential doublets were removed by applying the DoubletFinder (McGinnis et al., 2019) (RRID:SCR_018771) and DoubletDecon (Aran et al., 2019) packages with default parameters. Only the cells marked as doublets by both algorithms were removed. The remaining QC-pass cells were analyzed using the Seurat package (Satija et al., 2015) (RRID:SCR_016341) and batch-corrected using the Harmony package (Korsunsky et al., 2019). In brief, the single cells were normalized based on their library size and later log-transformed. For dimensionality reduction, principal component analysis was applied on the 2000 most variable genes and the first 30 computed PCs were used as an input for Leiden-based clustering. For cell type assignment, the cluster-specific marker genes were computed and visualized in dot plots or feature plots for cell type inference. In parallel, a supervised algorithm SingleR (Aran et al., 2019), that relies on pre-defined reference transcriptome profiles, was used to compare the single-cell transcriptomes and assign the cell type labels. The single-cell results, marker genes and overlaps with bulk RNA-seq were visualized using heatmaps, dot, violin, or radar plots in R language and Partek Genomics Suite (RRID:SCR_011860).

### 4.9. Enrichment analyses of differentially expressed genes from bulk and scRNA-seq

The functional enrichment analyses were performed using g:Profiler (RRID:SCR_006809) (Raudvere et al., 2019) and Mus musculus as the species. Differentially expressed genes were ranked by significance (FDR) and analyzed by g:SCS multiple testing correction method and significance threshold set to 0.05. Statistical significance was calculated using a custom background list of all genes expressed in the ovary as detected by bulk RNA sequencing. Functional analysis was conducted using the Gene Ontology database for molecular function (GO:MF), cellular component (GO:CC), and biological function (GO:BF) and KEGG database for biological pathways. Minimum and Maximum term size limits were set at 10 and 1000 genes.

Gene set enrichment analysis (GSEA) for bulk RNA-seq normalized gene expression data was performed using the GSEA software (GSEA version 4.2.3) (RRID:SCR 001905) (Subramanian et al., 2005) and the hallmark gene set collection (RRID:SCR_016863) from the Molecular Signatures Database (MsigDB) (Liberzon et al., 2011). The number of permutations was set to 1000 (geneset) and dataset minimum and maximum size were set to 10 and 1000, respectively. Absolute NES (normalized enrichment score) > 1.6 and FDR < 0.05 were set as cut-offs for significant enrichment. For scRNA-seq data, we used the fGSEA (RRID:SCR_020938) R package for pathway enrichment analysis and tested for different gene sets like Hallmarks gene sets from MSigDB database (Liberzon et al., 2011) (RRID:SCR_016863). For fGSEA input, genes were pre-ranked using a fast Wilcoxon rank-sum test (presto R package V1.0.0) to test the enrichment of different gene sets. Enrichment analysis for predicted transcription factors (TF) was conducted using g:Profiler and the TRANSFAC database (with no term size limit). Further TFs enrichment analysis was performed by ChEA3 (RRID:SCR_023159) with ReMap Chip-seq library (Keenan et al., 2019).

## Supplementary Material

Supp 1

Supp 2

Supp 3

Supp 4

Supp 5

Supplementary data to this article can be found online at https://doi.org/10.1016/j.ydbio.2024.09.007.

## Data Availability

Data will be made available on request.

## Data and code availability

- The bulk RNA-seq and scRNA-seq data from this study are available on GEO (accession number GSE255032)
- This paper does not report the original code.
- Any additional information required to reanalyze the data reported in this paper is available from the corresponding author upon request.
