# Supplementary material for: Single-cell and bulk transcriptional profiling of mouse ovaries reveals novel genes and pathways associated with DNA damage response in oocytes
Source: Dev Biol. Author manuscript; Available in PMC 2025 Sep 2. (PMC12403231; doi:10.1016/j.ydbio.2024.09.007)
Supplement: Supp 1 [file NIHMS2102123-supplement-Supp_1.docx]

**Single-cell and bulk transcriptional profiling of mouse ovaries reveals novel genes and pathways associated with DNA damage response in oocytes.**

Monique Mills^1,2^, Chihiro Emori^4^, Parveen Kumar^3^, Zachary Boucher^1^, Joshy George^3^ and Ewelina Bolcun-Filas^1^*

**Affiliations**

*1 The Jackson Laboratory, 600 Main Street, Bar Harbor, ME, 04609, USA.*

*2 The Graduate School of Biomedical Science and Engineering, University of Maine, Orono, ME, 04469, USA*

*3* *The Jackson Laboratory for Genomic Medicine, Farmington, CT, 06110, USA.*

*4 Department of Experimental Genome Research, Research Institute for Microbial Diseases, Osaka University, Suita, Osaka, 5650871, Japan*

*Corresponding author: [Ewelina.Bolcun-Filas@jax.org](mailto:Ewelina.Bolcun-Filas@jax.org)

Keywords: DNA Damage Response, CHEK2, oocyte, ovary, single-cell transcriptomics

**Supplementary Materials:**

**Supplementary Figures (S1-S9)**

**Supplementary Tables (S1-S4)**

**Figure S1.**


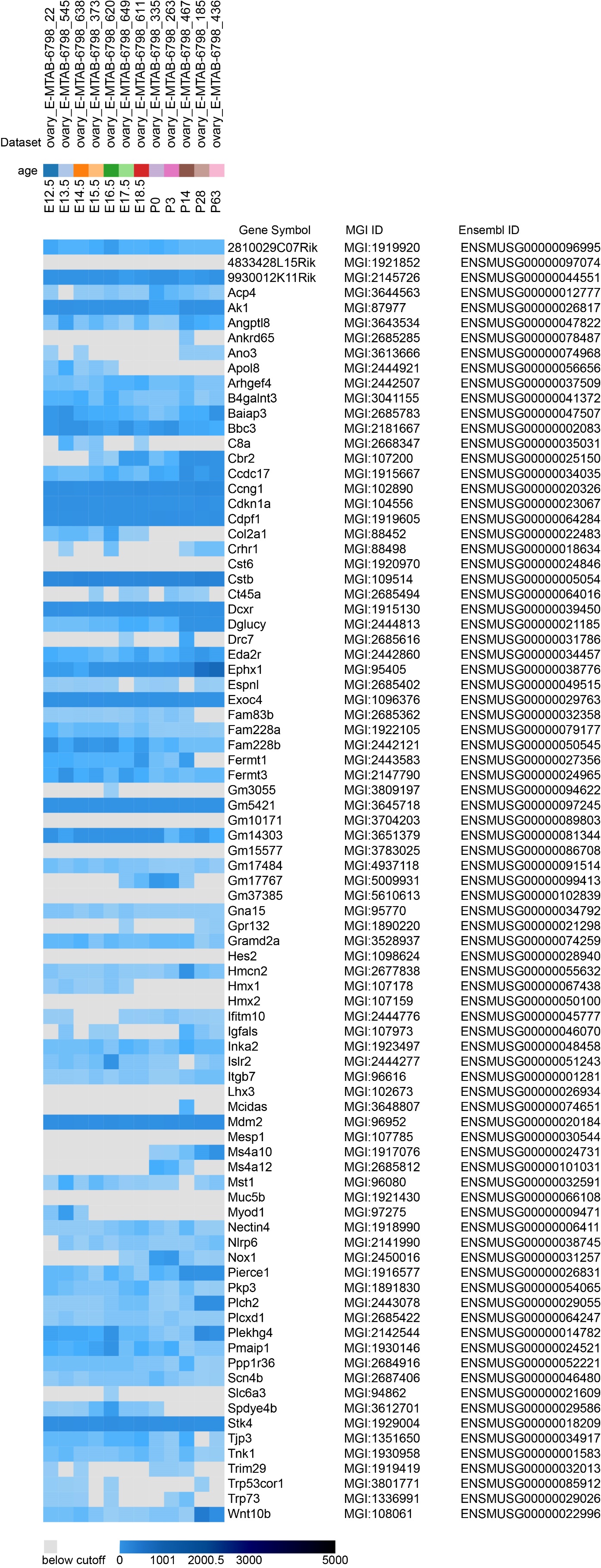


**Figure S1**. Expression of RRGs identified in the study during ovary development generated using published data and Gene Expression Database (GXD) at Mouse Genome Informatics Portal https://www.informatics.jax.org/expression.shtml

**Figure S2.**


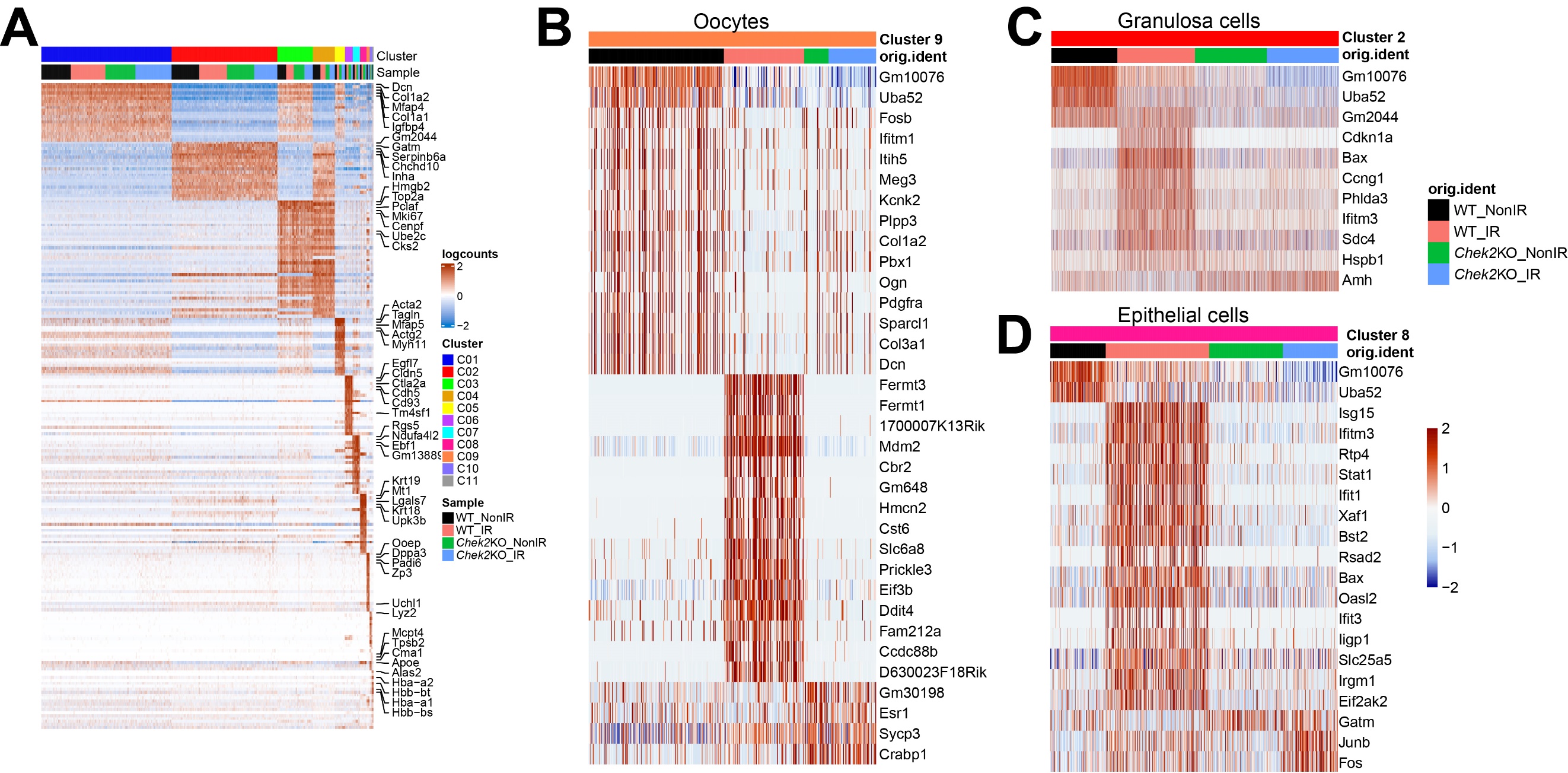
**Figure S2**. **A**) Heatmap of marker gene expression from single cells obtained from wild-type and *Chek2^-/-^* ovaries with and without radiation organized into 11 clusters. **B**-**D**) Heatmaps of differentially expressed genes in oocyte, granulosa, and epithelial cell clusters.

**Figure S3.**


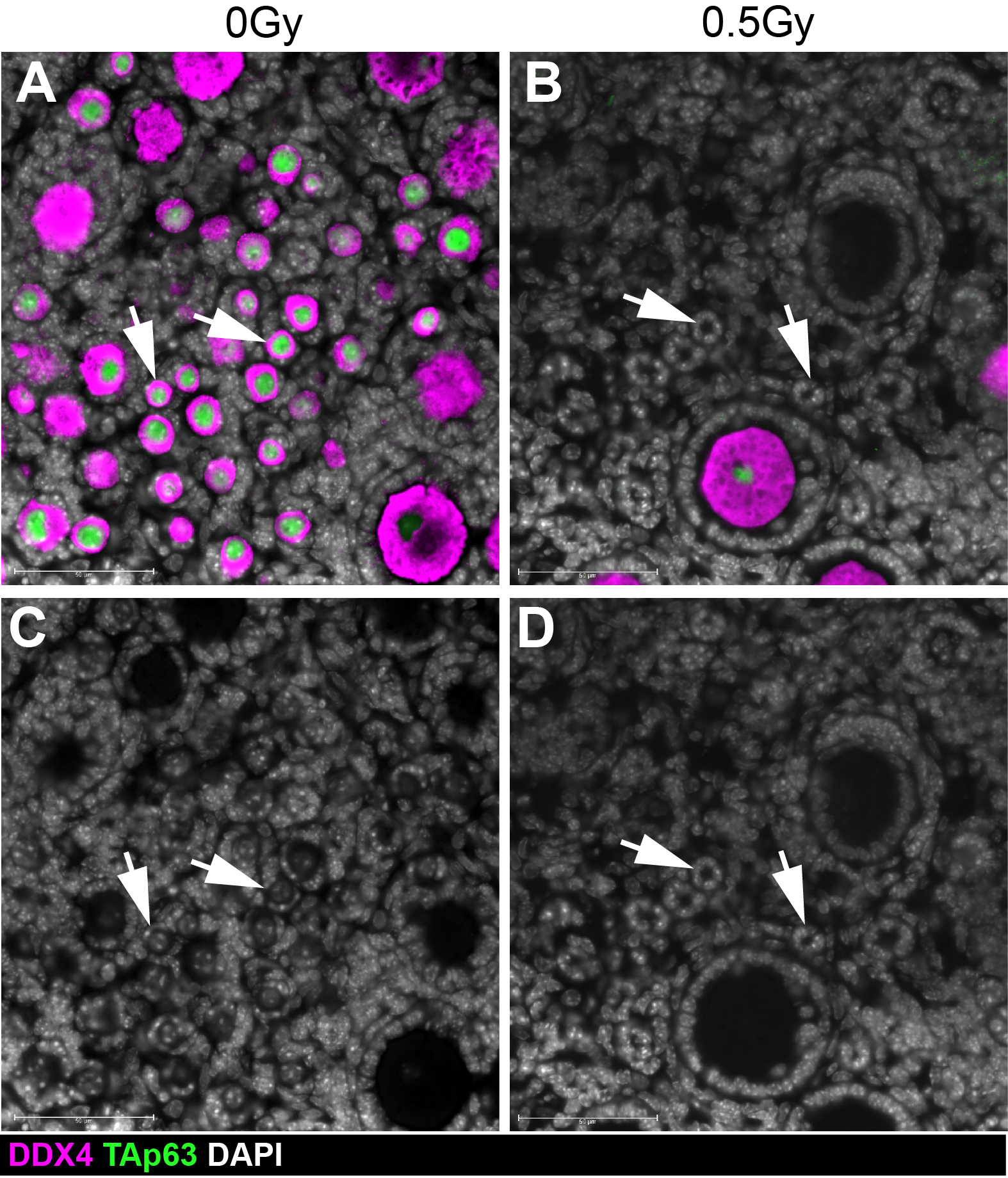


**Figure S3**. Example image of non-irradiated (**A**) and irradiated (**B**) ovaries immunostained with oocyte markers DDX4 and TAp63. **C-D)** Grayscale image for DAPI channel to visualize nuclei. Arrows indicate primordial follicles with an oocyte inside in non-irradiated (**C**) and empty follicles with visible granulosa cells but without the oocyte in the irradiated ovary (**D**).

**Figure S4.**


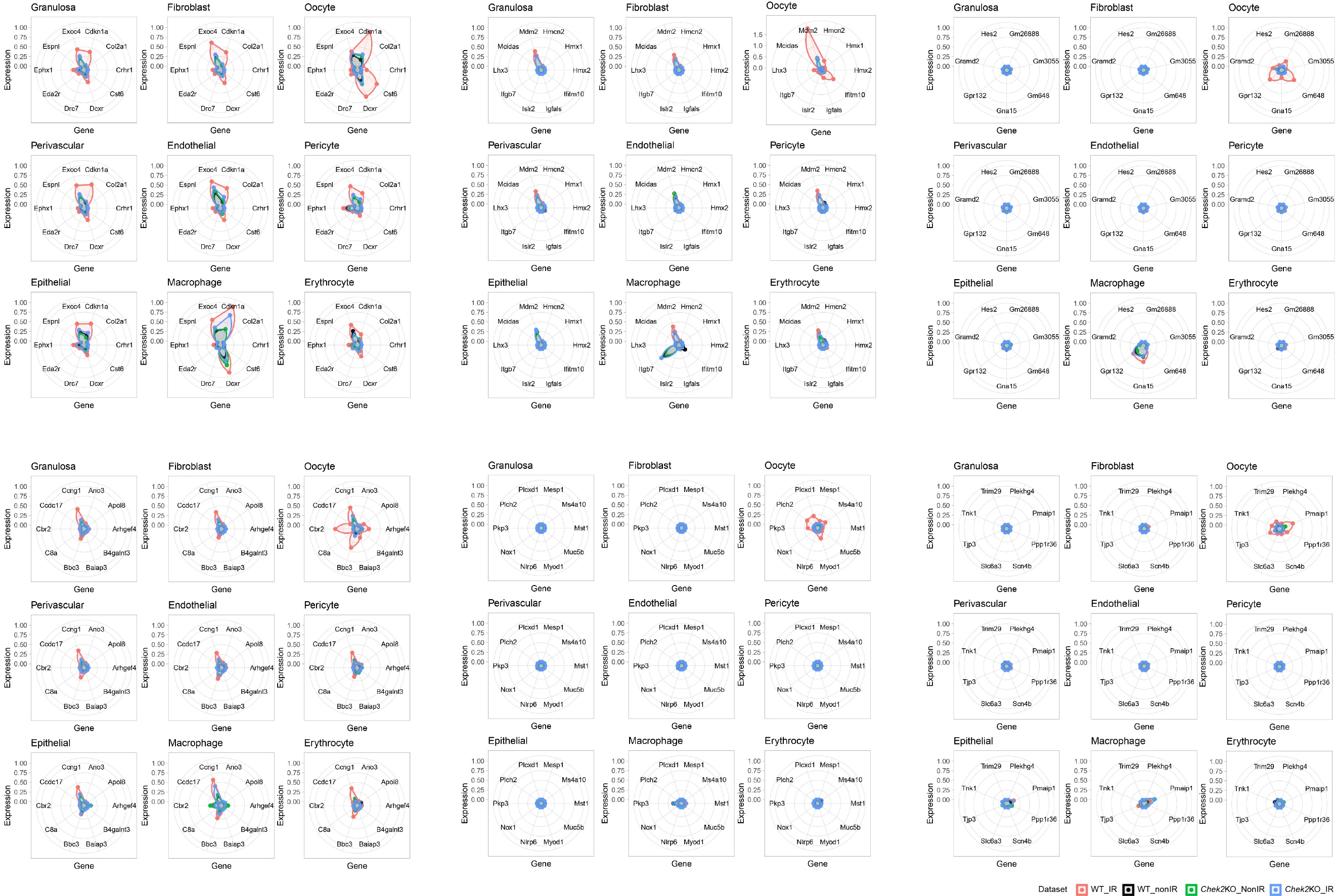


**Figure S4**. Radar charts showing expression of RRGs in different cell types. Each plot compares cells from wild-type irradiated (red), wild-type non-irradiated (black), *Chek2^-/-^* non-irradiated (green), and *Chek2^-/-^* irradiated (blue)

**Figure S5.**


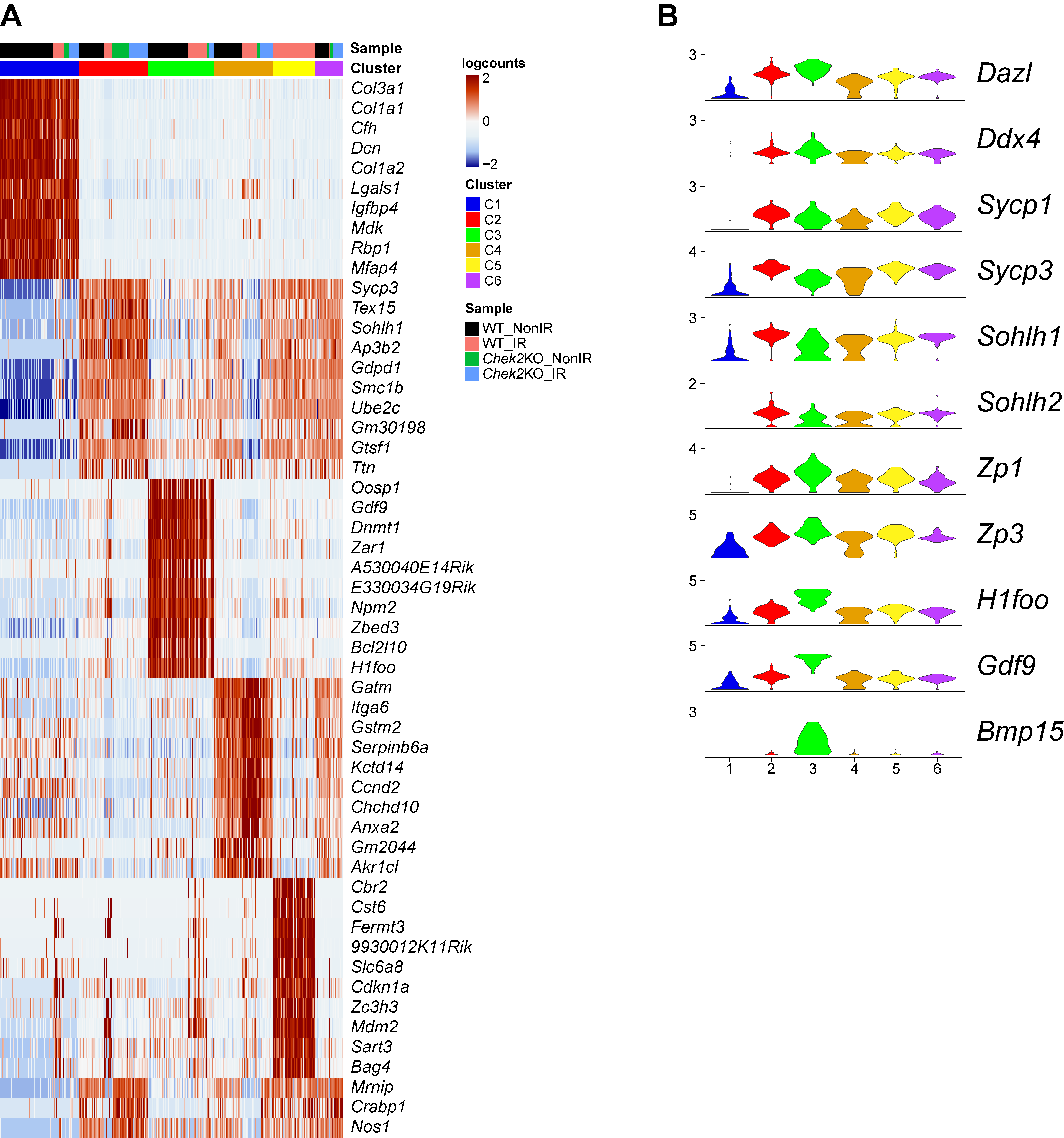


**Figure S5.** **A**) Heatmap displaying marker gene expression for six oocyte subclusters identified through *de novo* re-clustering. **B**) Violin plots illustrating the expression of key genes involved in oocyte development across the oocyte subclusters.

**Figure S6.**


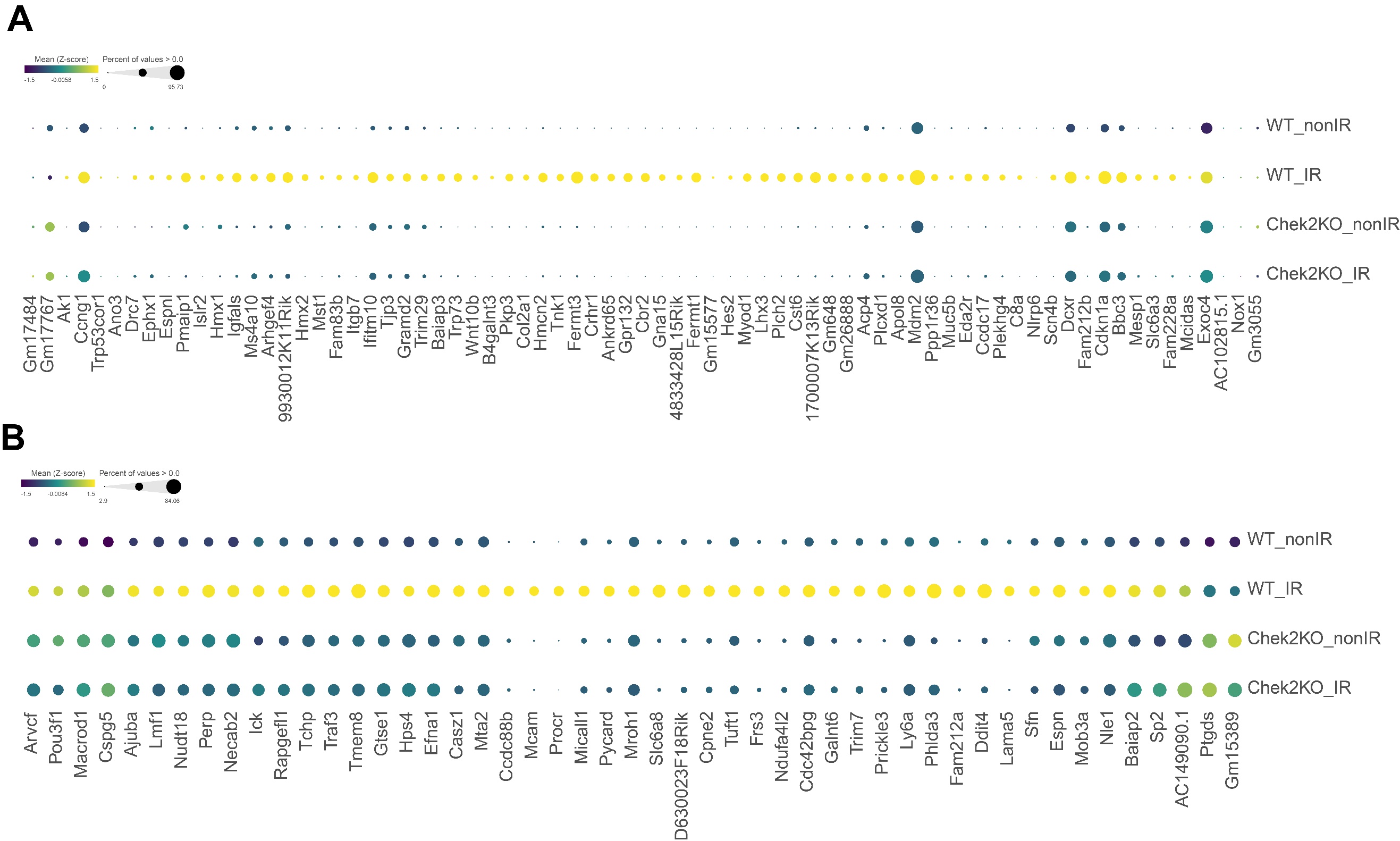


**Figure S6.** Bubble plot showing expression of RRGs from bulk **(A)** and additional RRGs from subcluster analysis **(B)** in all oocytes from wild-type and *Chek2^-/-^* ovaries with and without radiation. Bubble size is proportional to the percentage of cells in a cluster expressing a gene, and color intensity is proportional to average scaled gene expression within a cluster.

**Figure S7**


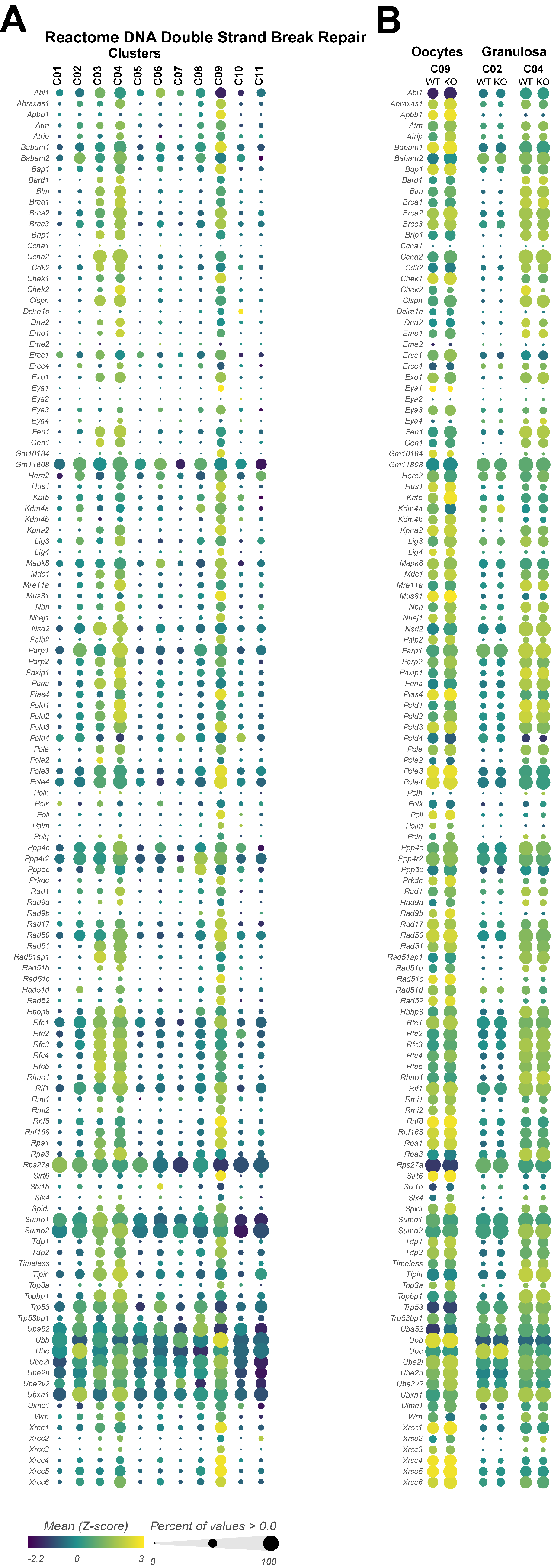


**Figure S7.** **A**) Bubble plot showing expression of 160 genes involved in DSB repair (Reactome Patwhays) across ovarian cell types in non-irradiated ovaries. (**B**) Bubble plot showing expression of DSB repair genes in wild-type and *Chek2^-/-^* non-irradiated oocytes and granulosa cells. Bubble size is proportional to the percentage of cells in a cluster expressing a gene, and color intensity is proportional to average scaled gene expression within a cluster.

**Figure S8.**


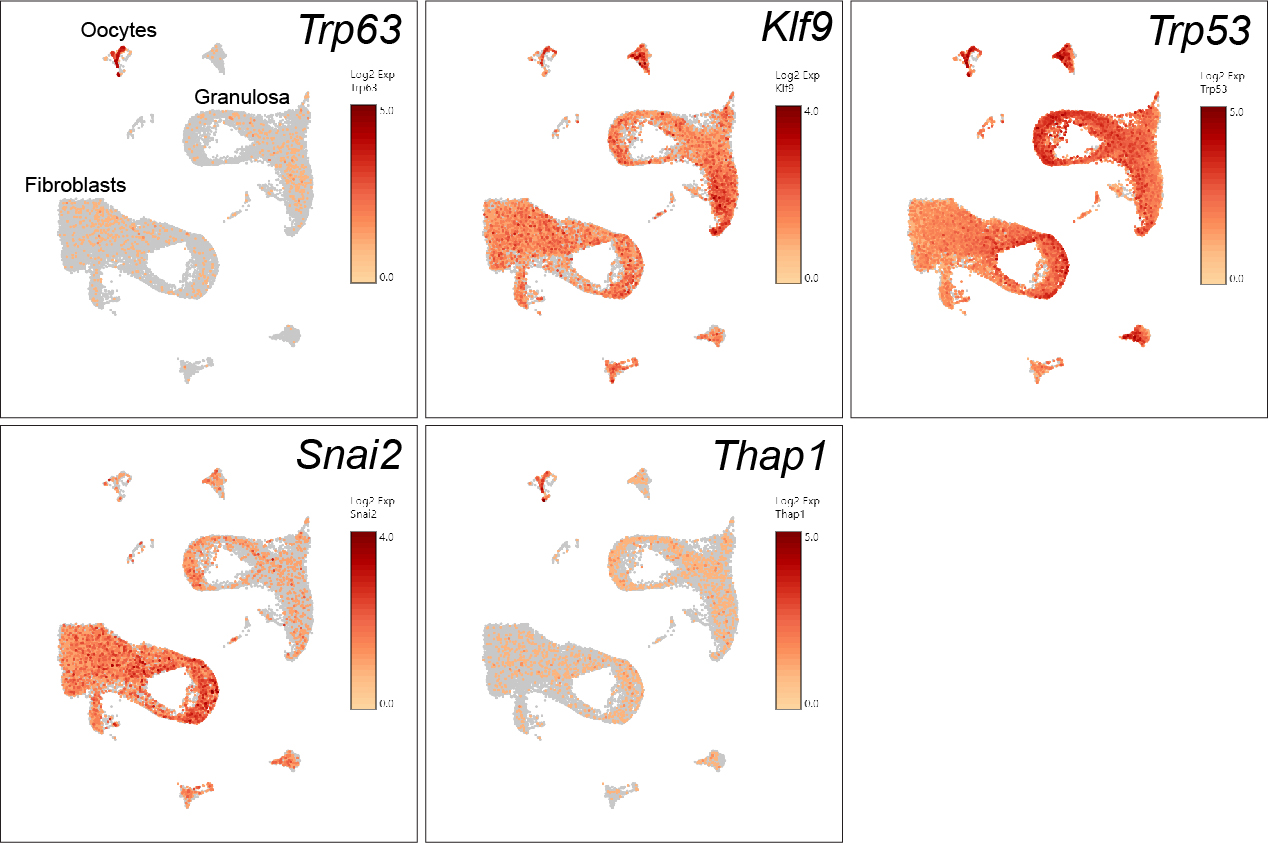


**Figure S8.** Gene expression levels of transcription factors predicted to regulate RRGs overlaid on UMAP plots with the oocyte, granulosa, and fibroblast clusters indicated.

**Figure S9.**


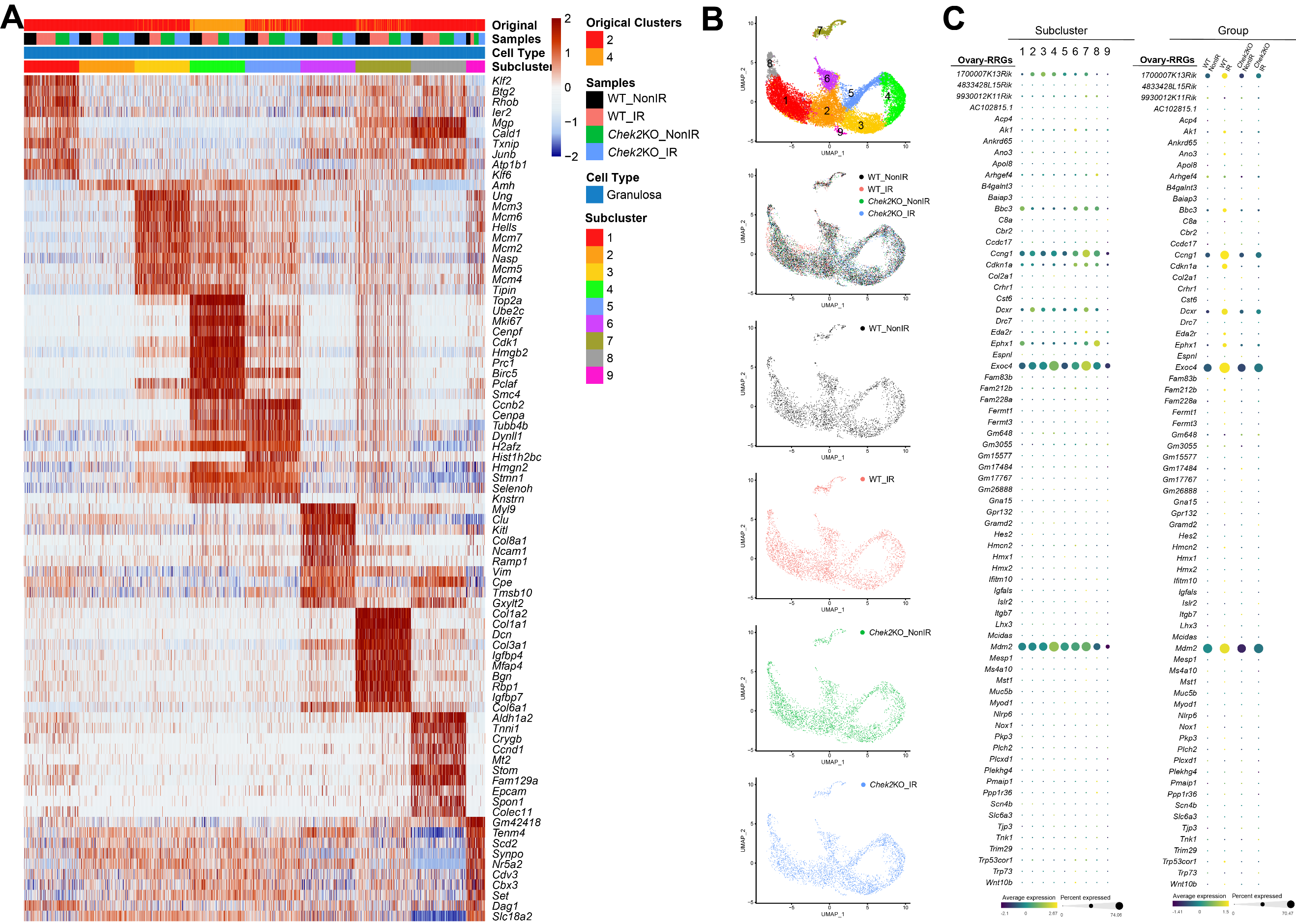


**Figure S9. A**) Heatmap displaying marker gene expression for nine granulosa cell subclusters identified through *de novo* re-clustering. **B**) UMAP plots of granulosa subclusters and contribution of cells from all treatment groups. **C**) Bubble plot showing expression of ovary-RRGs from bulk analysis across granulosa subclusters and in granulosa cells from wild-type and *Chek2^-/-^* ovaries with and without radiation. Bubble size is proportional to the percentage of cells in a cluster expressing a gene, and color intensity is proportional to average scaled gene expression within a cluster.

**Supplementary Tables:**

**Supplementary Table S1. RRGs identified by bulk RNA-seq.**

| Table S1. Radiation Responsive Genes in the ovary  Part 1. Response in wildtype ovaries. | | | | | |  |
| --- | --- | --- | --- | --- | --- | --- |
| MGI Gene/ Marker ID | **Symbol** | **LogFC WT** | **Adj p-value** | **Feature Type** | **Gene Ontology terms** | |
| MGI:5610613 | [*Gm37385*](http://www.informatics.jax.org/marker/MGI:5610613) | 22.7 | 8.77E-05 | unclassified gene |  | |
| MGI:3809197 | [*Gm3055*](http://www.informatics.jax.org/marker/MGI:3809197) | 20.6 | 1.02E-03 | protein coding gene |  | |
| MGI:102673 | [*Lhx3*](http://www.informatics.jax.org/marker/MGI:102673) | 7.8 | 1.14E-10 | protein coding gene | cell differentiation; DNA-binding transcription factor activity, RNA polymerase II-specific | |
| MGI:1921852 | [*4833428L15Rik*](http://www.informatics.jax.org/marker/MGI:1921852) | 6.9 | 1.06E-07 | lncRNA gene |  | |
| MGI:2685285 | [*Ankrd65*](http://www.informatics.jax.org/marker/MGI:2685285) | 6.7 | 1.22E-18 | protein coding gene |  | |
| MGI:107159 | [*Hmx2*](http://www.informatics.jax.org/marker/MGI:107159) | 6.5 | 3.27E-05 | protein coding gene | cell differentiation; DNA-binding transcription factor activity, RNA polymerase II-specific | |
| MGI:2444921 | [*Apol8*](http://www.informatics.jax.org/marker/MGI:2444921) | 5.8 | 6.63E-08 | protein coding gene | chloride channel activity; extracellular space; high-density lipoprotein particle; intracellular membrane-bounded organelle | |
| No associated gene | *Gm43301* | 5.7 | 1.22E-04 | lncRNA gene |  | |
| MGI:3613666 | [*Ano3*](http://www.informatics.jax.org/marker/MGI:3613666) | 5.6 | 1.27E-30 | protein coding gene | calcium activated phospholipid scrambling; chloride channel activity; chloride transmembrane transport | |
| MGI:3783025 | [*Gm15577*](http://www.informatics.jax.org/marker/MGI:3783025) | 5.5 | 5.92E-03 | lncRNA gene |  | |
| MGI:107785 | [*Mesp1*](http://www.informatics.jax.org/marker/MGI:107785) | 5.5 | 2.99E-03 | protein coding gene | DNA-binding transcription activator activity, RNA polymerase II-specific; Notch signaling pathway | |
| MGI:1098624 | [*Hes2*](http://www.informatics.jax.org/marker/MGI:1098624) | 5.5 | 6.45E-08 | protein coding gene | DNA-binding transcription factor activity, RNA polymerase II-specific; DNA-binding transcription repressor activity, RNA polymerase II-specific | |
| MGI:2443078 | [*Plch2*](http://www.informatics.jax.org/marker/MGI:2443078) | 5.3 | 8.57E-51 | protein coding gene | calcium ion binding; intracellular signal transduction; phosphatidylinositol-mediated signaling; phospholipase C activity; plasma membrane; signal transduction | |
| MGI:94862 | [*Slc6a3*](http://www.informatics.jax.org/marker/MGI:94862) | 5.2 | 2.50E-11 | protein coding gene | cell surface; dopamine transport; integral component of membrane; signaling receptor binding | |
| MGI:107200 | [*Cbr2*](http://www.informatics.jax.org/marker/MGI:107200) | 5.1 | 2.91E-05 | protein coding gene | carbonyl reductase (NADPH) activity; glucose metabolic process; mitochondrion; oxidoreductase activity | |
| MGI:97275 | [*Myod1*](http://www.informatics.jax.org/marker/MGI:97275) | 4.8 | 6.15E-07 | protein coding gene | cell differentiation; cellular response to estradiol stimulus; cellular response to oxygen levels; cellular response to tumor necrosis factor; DNA-binding transcription activator activity, RNA polymerase II-specific; ubiquitin protein ligase binding | |
| MGI:88498 | [*Crhr1*](http://www.informatics.jax.org/marker/MGI:88498) | 4.7 | 2.07E-09 | protein coding gene | adenylate cyclase-activating G protein-coupled receptor; signaling pathway; cell surface receptor signaling pathway; endosome; transmembrane signaling receptor activity; vesicle | |
| MGI:2685402 | [*Espnl*](http://www.informatics.jax.org/marker/MGI:2685402) | 4.7 | 2.42E-18 | protein coding gene | actin binding; cell projection; stereocilium tip; stereocilium tip | |
| MGI:2668347 | [*C8a*](http://www.informatics.jax.org/marker/MGI:2668347) | 4.7 | 1.39E-03 | protein coding gene | complement activation; cytolysis; immune response; membrane attack complex | |
| MGI:2443583 | [*Fermt1*](http://www.informatics.jax.org/marker/MGI:2443583) | 4.5 | 9.82E-06 | protein coding gene | cell adhesion; cell junction; focal adhesion; integrin-mediated signaling pathway; negative regulation of; canonical Wnt signaling pathway; positive regulation of cell adhesion mediated by integrin | |
| MGI:1890220 | [*Gpr132*](http://www.informatics.jax.org/marker/MGI:1890220) | 4.5 | 1.42E-15 | protein coding gene | G1/S transition of mitotic cell cycle; G protein-coupled receptor signaling pathway; negative regulation of G2/M; transition of mitotic cell cycle; plasma membrane; signal transduction | |
| MGI:2685616 | [*Drc7*](http://www.informatics.jax.org/marker/MGI:2685616) | 4.4 | 5.93E-07 | protein coding gene | cell differentiation; cell projection; cilium | |
| MGI:88452 | [*Col2a1*](http://www.informatics.jax.org/marker/MGI:88452) | 4.3 | 1.35E-06 | protein coding gene | cellular response to BMP stimulus; extracellular region | |
| MGI:3648807 | [*Mcidas*](http://www.informatics.jax.org/marker/MGI:3648807) | 4.2 | 2.60E-02 | protein coding gene | cell cycle; negative regulation of cell cycle; negative regulation of DNA replication | |
| MGI:2444776 | [*Ifitm10*](http://www.informatics.jax.org/marker/MGI:2444776) | 4.2 | 5.11E-37 | protein coding gene | integral component of membrane; membrane | |
| MGI:1916577 | [*Pierce1*](http://www.informatics.jax.org/marker/MGI:1916577) *(1700007K13Rik)* | 4.1 | 1.10E-18 | protein coding gene | cellular response to DNA damage stimulus; regulation of gene expression | |
| MGI:2685494 | [*Gm648*](http://www.informatics.jax.org/marker/MGI:2685494) | 4.1 | 2.26E-10 | protein coding gene |  | |
| MGI:95770 | [*Gna15*](http://www.informatics.jax.org/marker/MGI:95770) | 4.0 | 3.05E-05 | protein coding gene | adenylate cyclase-modulating G protein-coupled receptor signaling pathway; GTPase activity membrane; phospholipase C-activating G protein-coupled receptor signaling pathway | |
| MGI:1918990 | [*Nectin4*](http://www.informatics.jax.org/marker/MGI:1918990) *(Pvrl4)* | 3.9 | 3.12E-10 | protein coding gene | cell junction | |
| MGI:1919920 | [*2810029C07Rik*](http://www.informatics.jax.org/marker/MGI:1919920) | 3.9 | 9.10E-37 | lncRNA gene |  | |
| MGI:2444277 | [*Islr2*](http://www.informatics.jax.org/marker/MGI:2444277) | 3.8 | 2.02E-11 | protein coding gene | cell surface; integral component of membrane; protein binding | |
| MGI:2687406 | [*Scn4b*](http://www.informatics.jax.org/marker/MGI:2687406) | 3.7 | 2.07E-11 | protein coding gene | ion transport; plasma membrane; sodium channel activity | |
| MGI:2684916 | [*Ppp1r36*](http://www.informatics.jax.org/marker/MGI:2684916) | 3.7 | 1.11E-09 | protein coding gene | negative regulation of phosphatase activity; phosphatase binding | |
| MGI:3612701 | [*Spdye4b*](http://www.informatics.jax.org/marker/MGI:3612701) *(4933411G11Rik)* | 3.7 | 3.15E-03 | protein coding gene | protein kinase binding | |
| MGI:2147790 | [*Fermt3*](http://www.informatics.jax.org/marker/MGI:2147790) | 3.6 | 4.15E-10 | protein coding gene | cell adhesion; cell junction; integrin-mediated signaling pathway | |
| MGI:3645718 | [*Gm5421*](http://www.informatics.jax.org/marker/MGI:3645718) | 3.6 | 1.67E-02 | pseudogene |  | |
| MGI:2685362 | [*Fam83b*](http://www.informatics.jax.org/marker/MGI:2685362) | 3.3 | 3.85E-23 | protein coding gene | phosphatidylinositol 3-kinase catalytic subunit binding; signal transduction | |
| MGI:1921430 | [*Muc5b*](http://www.informatics.jax.org/marker/MGI:1921430) | 3.3 | 4.79E-08 | protein coding gene | extracellular matrix; regulation of macrophage activation | |
| MGI:2444813 | [*Dglucy*](http://www.informatics.jax.org/marker/MGI:2444813) *(9030617O03Rik)* | 3.3 | 5.40E-16 | protein coding gene | D-glutamate cyclase activity; lyase activity; mitochondrion | |
| MGI:107178 | [*Hmx1*](http://www.informatics.jax.org/marker/MGI:107178) | 3.2 | 2.56E-05 | protein coding gene | DNA-binding transcription factor activity, RNA polymerase II-specific | |
| MGI:3801771 | [*Trp53cor1*](http://www.informatics.jax.org/marker/MGI:3801771) | 3.2 | 2.03E-04 | lncRNA gene | negative regulation of gene expression; ribonucleoprotein complex | |
| MGI:1920970 | [*Cst6*](http://www.informatics.jax.org/marker/MGI:1920970) | 3.2 | 4.08E-04 | protein coding gene | epidermis development | |
| MGI:1930958 | [*Tnk1*](http://www.informatics.jax.org/marker/MGI:1930958) | 3.0 | 7.58E-06 | protein coding gene | ATP binding; kinase activity; negative regulation of Ras protein signal transduction; transmembrane receptor protein tyrosine kinase signaling pathway | |
| MGI:108061 | [*Wnt10b*](http://www.informatics.jax.org/marker/MGI:108061) | 3.0 | 1.04E-02 | protein coding gene | canonical Wnt signaling pathway; G2/M transition of mitotic cell cycle; negative regulation of transcription by RNA polymerase II; regulation of cell cycle | |
| MGI:96080 | [*Mst1*](http://www.informatics.jax.org/marker/MGI:96080) | 3.0 | 4.59E-04 | protein coding gene | extracellular region; negative regulation of epithelial cell apoptotic process; receptor tyrosine kinase binding; regulation of receptor signaling pathway via JAK-STAT serine-type endopeptidase activity | |
| MGI:1915667 | [*Ccdc17*](http://www.informatics.jax.org/marker/MGI:1915667) | 3.0 | 7.06E-18 | protein coding gene |  | |
| MGI:5477382 | [*Gm26888*](http://www.informatics.jax.org/marker/MGI:5477382) | 2.8 | 4.60E-09 | lncRNA gene |  | |
| MGI:1336991 | [*Trp73*](http://www.informatics.jax.org/marker/MGI:1336991) | 2.8 | 8.25E-11 | protein coding gene | apoptotic process; cell cycle; cell junction; cellular response to DNA damage stimulus; DNA-binding transcription activator activity, RNA polymerase II-specific; intrinsic apoptotic signaling pathway in response to DNA damage by p53 class mediator; MDM2/MDM4 family protein binding; negative regulation of JUN kinase activity; p53 binding; positive regulation of apoptotic process; positive regulation of MAPK cascade | |
| MGI:1930146 | [*Pmaip1*](http://www.informatics.jax.org/marker/MGI:1930146) | 2.7 | 1.16E-16 | protein coding gene | activation of cysteine-type endopeptidase activity; involved in apoptotic process; positive regulation of apoptotic process; positive regulation of DNA damage response, signal transduction by p53 class mediator; reactive oxygen species metabolic process; regulation of apoptotic process | |
| MGI:3643534 | [*Angptl8*](http://www.informatics.jax.org/marker/MGI:3643534) *(Gm6484)* | 2.6 | 3.32E-04 | protein coding gene | extracellular region; negative regulation of lipoprotein lipase activity | |
| MGI:1891830 | [*Pkp3*](http://www.informatics.jax.org/marker/MGI:1891830) | 2.6 | 6.08E-07 | protein coding gene | adherens junction; alpha-catenin binding; cell-cell adhesion; cell-cell junction assembly | |
| MGI:2142544 | [*Plekhg4*](http://www.informatics.jax.org/marker/MGI:2142544) | 2.6 | 6.96E-03 | protein coding gene | activation of GTPase activity | |
| MGI:1351650 | [*Tjp3*](http://www.informatics.jax.org/marker/MGI:1351650) | 2.5 | 4.90E-06 | protein coding gene | cell-cell adhesion; cell-cell junction; cell surface; regulation of G1/S transition of mitotic cell cycle | |
| MGI:3644563 | [*Acp4*](http://www.informatics.jax.org/marker/MGI:3644563)*(Acpt)* | 2.5 | 1.09E-07 | protein coding gene | acid phosphatase activity; negative regulation of ERBB4 signaling pathway; peptidyl-tyrosine dephosphorylation involved in inactivation of protein kinase activity; protein tyrosine phosphatase activity | |
| MGI:96616 | [*Itgb7*](http://www.informatics.jax.org/marker/MGI:96616) | 2.4 | 2.61E-04 | protein coding gene | cell adhesion; cell adhesion mediated by integrin; focal adhesion; integrin-mediated signaling pathway | |
| MGI:1919419 | [*Trim29*](http://www.informatics.jax.org/marker/MGI:1919419) | 2.4 | 5.51E-03 | protein coding gene | innate immune response; lysosome; negative regulation of transcription by RNA polymerase II; p53 binding | |
| MGI:1922105 | [*Fam228a*](http://www.informatics.jax.org/marker/MGI:1922105) | 2.4 | 1.30E-02 | protein coding gene |  | |
| MGI:3528937 | [*Gramd2*](http://www.informatics.jax.org/marker/MGI:3528937) | 2.3 | 5.77E-08 | protein coding gene | extrinsic component of cytoplasmic side of plasma membrane; integral component of membrane; phosphatidylinositol binding | |
| MGI:1917076 | [*Ms4a10*](http://www.informatics.jax.org/marker/MGI:1917076) | 2.1 | 2.42E-03 | protein coding gene | cell surface receptor signaling pathway; integral component of membrane; membrane | |
| MGI:4937118 | [*Gm17484*](http://www.informatics.jax.org/marker/MGI:4937118) | 2.1 | 5.54E-05 | lncRNA gene |  | |
| MGI:2145726 | [*9930012K11Rik*](http://www.informatics.jax.org/marker/MGI:2145726) | 2.1 | 1.26E-12 | protein coding gene |  | |
| MGI:102890 | [*Ccng1*](http://www.informatics.jax.org/marker/MGI:102890) | 2.0 | 5.64E-105 | protein coding gene | cell cycle; cell division; cyclin-dependent protein; serine/threonine kinase regulator activity; cytoplasm; mitotic G2 DNA damage checkpoint signaling; negative regulation of apoptotic process | |
| MGI:87977 | [*Ak1*](http://www.informatics.jax.org/marker/MGI:87977) | 2.0 | 1.64E-05 | protein coding gene | adenylate kinase activity; regulation of G1/S transition of mitotic cell cycle | |
| MGI:2685783 | [*Baiap3*](http://www.informatics.jax.org/marker/MGI:2685783) | 2.0 | 1.95E-05 | protein coding gene | calcium ion binding; G protein-coupled receptor signaling pathway; late endosome membrane; regulation of dense core granule exocytosis | |
| MGI:2685422 | [*Plcxd1*](http://www.informatics.jax.org/marker/MGI:2685422) | 1.9 | 1.10E-07 | protein coding gene | lipid metabolic process | |
| MGI:2181667 | [*Bbc3*](http://www.informatics.jax.org/marker/MGI:2181667) | 1.9 | 3.41E-11 | protein coding gene | activation of cysteine-type endopeptidase activity; involved in apoptotic process; cellular response to DNA damage stimulus; cellular response to ionizing radiation; intrinsic apoptotic signaling pathway; intrinsic apoptotic signaling pathway by p53 class mediator; PUMA-BCL-xl complex; response to endoplasmic reticulum stress | |
| MGI:107973 | [*Igfals*](http://www.informatics.jax.org/marker/MGI:107973) | 1.9 | 2.92E-02 | protein coding gene | cell adhesion; extracellular region | |
| MGI:2677838 | [*Hmcn2*](http://www.informatics.jax.org/marker/MGI:2677838) | 1.8 | 1.92E-03 | protein coding gene | basement membrane; cell junction; collagen-containing extracellular matrix; response to stimulus | |
| MGI:1923497 | [*Inka2*](http://www.informatics.jax.org/marker/MGI:1923497) *(Fam212b)* | 1.8 | 2.95E-13 | protein coding gene | negative regulation of catalytic activity; protein serine/threonine kinase inhibitor activity | |
| MGI:2442860 | [*Eda2r*](http://www.informatics.jax.org/marker/MGI:2442860) | 1.8 | 2.19E-21 | protein coding gene | cell differentiation; integral component of membrane; intrinsic apoptotic signaling pathway by p53 class mediator; positive regulation of I-kappaB kinase/NF-kappaB signaling; positive regulation of JNK cascade programmed cell death; tumor necrosis factor-mediated signaling pathway | |
| MGI:3041155 | [*B4galnt3*](http://www.informatics.jax.org/marker/MGI:3041155) | 1.7 | 3.26E-03 | protein coding gene | acetylgalactosaminyltransferase activity; glycosyltransferase activity; Golgi apparatus | |
| MGI:95405 | [*Ephx1*](http://www.informatics.jax.org/marker/MGI:95405) | 1.7 | 3.26E-03 | protein coding gene | integral component of membrane; plasma membrane; response to toxic substance | |
| MGI:104556 | [*Cdkn1a*](http://www.informatics.jax.org/marker/MGI:104556) | 1.7 | 5.54E-05 | protein coding gene | cell cycle; cellular response to DNA damage stimulus; cellular response to ionizing radiation; cellular response to UV-B; cyclin-dependent protein serine/threonine kinase inhibitor activity; DNA damage response, signal; transduction by p53 class mediator resulting in cell cycle arrest; mitotic G2 DNA damage checkpoint signaling; PCNA-p21 complex; regulation of cell cycle G1/S phase transition | |
| MGI:1915130 | [*Dcxr*](http://www.informatics.jax.org/marker/MGI:1915130) | 1.6 | 2.91E-03 | protein coding gene | carbonyl reductase (NADPH) activity; L-xylulose reductase (NADP+) activity; membrane; oxidoreductase activity; positive regulation of reactive oxygen species; metabolic process | |
| MGI:2442507 | [*Arhgef4*](http://www.informatics.jax.org/marker/MGI:2442507) | 1.5 | 6.22E-04 | protein coding gene | intracellular signal transduction; lamellipodium assembly; protein domain specific binding | |
| MGI:1096376 | [*Exoc4*](http://www.informatics.jax.org/marker/MGI:1096376) | 1.4 | 9.61E-06 | protein coding gene | cell projection; exocytosis; regulation of protein transport; small GTPase binding; vesicle tethering involved in exocytosis | |
| MGI:96952 | [*Mdm2*](http://www.informatics.jax.org/marker/MGI:96952) | 1.3 | 3.40E-07 | protein coding gene | apoptotic process; cellular response to gamma radiation; DNA damage response, signal transduction by p53 class mediator resulting in cell cycle arrest; NEDD8 ligase activity; p53 binding | |
| MGI:5009931 | [*Gm17767*](http://www.informatics.jax.org/marker/MGI:5009931) | -2.2 | 4.93E-03 | lncRNA gene |  | |
| MGI:2450016 | [*Nox1*](http://www.informatics.jax.org/marker/MGI:2450016) | -2.6 | 7.06E-05 | protein coding gene | cell junction; cellular response to hyperoxia; endosome; extracellular matrix organization; NADPH oxidase complex; oxidoreductase activity; positive regulation of JNK cascade; positive regulation of MAPK cascade; positive regulation of oxidative stress-induced intrinsic; apoptotic signaling pathway; small GTPase binding | |
| MGI:2141990 | [*Nlrp6*](http://www.informatics.jax.org/marker/MGI:2141990) | -3.1 | 8.69E-03 | protein coding gene | activation of cysteine-type endopeptidase activity; acute inflammatory response; ATP binding; inflammasome complex; necroptotic process; negative regulation of ERK1 and ERK2 cascade; negative regulation of I-kappaB kinase/NF-kappaB signaling; NLRP6 inflammasome complex | |
| No associated gene | *AC102815.1* | -15.2 | 1.96E-02 | unclassified gene |  | |
| MGI:3704203 | [*Gm10171*](http://www.informatics.jax.org/marker/MGI:3704203) | -20.2 | 1.64E-07 | pseudogene |  | |
| MGI:3651379 | [*Gm14303*](http://www.informatics.jax.org/marker/MGI:3651379) | -22.2 | 1.71E-04 | pseudogene |  | |

| Part 2. Response in *Chek2-/-* ovaries. | | | | | |
| --- | --- | --- | --- | --- | --- |
| MGI Gene/ Marker ID | **Symbol** | **LogFC *Chek2-/-*** | **Adj p-value** | **Feature Type** | **Gene Ontology terms** |
| MGI:5610613 | *Gm37385* | 26.4 | 1.77E-05 | unclassified gene |  |
| MGI:1338893 | *Padi1* | -6.7 | 0.032901 | protein coding gene | calcium ion binding; hydrolase activity; protein-arginine deiminase activity |
| MGI:102673 | *Gm43520* | -22.9 | 1.77E-05 | unclassified gene |  |

**Supplementary Table S2. Gene enrichment analysis of RRGs with g:Profiler.**

| Source | Term Name | Term ID | Adj.pvalue | -log10 adj.pvalue | Genes |
| --- | --- | --- | --- | --- | --- |
| GO:BP | intrinsic apoptotic signaling pathway by p53 class mediator | GO:0072332 | 0.001 | 3.15 | *Eda2r, Pmaip1, Bbc3, Trp73, Mdm2, Cdkn1a* |
| GO:BP | signal transduction by p53 class mediator | GO:0072331 | 0.019 | 1.71 | *Eda2r, Pmaip1, Bbc3, Trp73, Mdm2, Cdkn1a* |
| KEGG | p53 signaling pathway | KEGG:04115 | 0.000 | 6.62 | *Ccng1, Pmaip1, Bbc3, Trp73, Mdm2, Cdkn1a* |
| KEGG | Platinum drug resistance | KEGG:01524 | 0.001 | 3.05 | *Pmaip1, Bbc3, Mdm2, Cdkn1a* |
| KEGG | Apoptosis - multiple species | KEGG:04215 | 0.013 | 1.88 | *Pmaip1, Bbc3* |
| KEGG | Human papillomavirus infection | KEGG:05165 | 0.024 | 1.62 | *Hes2, Mdm2, Col2a1, Cdkn1a, Itgb7, Wnt10b* |
| KEGG | Colorectal cancer | KEGG:05210 | 0.036 | 1.44 | *Pmaip1, Bbc3, Cdkn1a* |
| WP | p53 signaling | WP:WP2902 | 0.000 | 7.07 | *Ccng1, Pmaip1, Bbc3, Trp73, Mdm2, Cdkn1a* |
| WP | Apoptosis | WP:WP1254 | 0.004 | 2.41 | *Pmaip1, Trp73, Mdm2* |
| WP | Hypoxia-dependent self-renewal of myoblasts | WP:WP5023 | 0.009 | 2.04 | *Myod1, Cdkn1a* |
| MIRNA | mmu-miR-23a-3p | MIRNA:mmu-miR-23a-3p | 0.030 | 1.52 | *Pmaip1, Bbc3* |

**Supplementary Table S3. Gene Set Enrichment Analysis of RRGs (GSEA).**

| HALLMARK PATHWAY | ES | NES | NOM p-val | FDR q-val | TOP GENES (HUMAN SYMBOLS) |
| --- | --- | --- | --- | --- | --- |
| P53 PATHWAY | 0.67 | 2.20 | 0.00 | 0.00 | *CCNG1, AK1, CLCA2, CDKN1A, DCXR, EPHX1, MDM2, PIDD1, PHLDA3, BTG2, ZMAT3, ZNF365, GLS2, MAPKAPK3, EPHA2, SFN, BAX, SLC19A2, DEF6, DDIT4* |
| INTERFERON ALPHA RESPONSE | 0.67 | 2.05 | 0.00 | 0.00 | *DHX58, RTP4, TRIM14, IRF9, LGALS3BP, TMEM140, TAP1, EIF2AK2, SAMD9L, PARP9, IRF7, HELZ2, PROCR, DDX60, TRIM21, ISG15, PSMB8, IFITM3, GBP4, HERC6* |
| INTERFERON GAMMA RESPONSE | 0.57 | 1.86 | 0.00 | 0.00 | *CDKN1A, ITGB7, DHX58, OAS2, RTP4, TRIM14, IRF9, LGALS3BP, TAP1, EIF2AK2, SAMD9L, DDX58, XAF1, STAT1, IRF7, HELZ2, DDX60, CSF2RB, TRIM21, ISG15* |
| APOPTOSIS | 0.53 | 1.70 | 0.00 | 0.01 | *PMAIP1, GNA15, CDKN1A, BTG2, BAX, TAP1, CD14, GUCY2D, IFITM3, CASP7, MMP2, GADD45A, IER3, CASP1, PLCB2, GSR, FAS, HGF, DPYD, TNFSF10* |
| TNFα SIGNALING VIA NFKB | 0.51 | 1.69 | 0.00 | 0.01 | *CDKN1A, BTG2, TAP1, EGR2, KYNU, DDX58, EGR1, DRAM1, LIF, F3, DUSP2, PLEK, PTGER4, EFNA1, IFIH1, LAMB3, SOCS3, IL7R, FOS, PLK2* |
| INFLAMMATORY RESPONSE | 0.49 | 1.61 | 0.00 | 0.02 | *GPR132, GNA15, CDKN1A, BTG2, RTP4, EIF2AK2, IRF7, LIF, RNF144B, BEST1, F3, CD14, ADORA2B, PTGER4, P2RX7, SEMA4D, HRH1, IL7R, ITGB8, CD48* |
| IL6-JAK/STAT3 SIGNALING | 0.52 | 1.56 | 0.01 | 0.03 | *IRF9, STAT1, IL1R2, CSF2RB, CD14, CCR1, SOCS3, CXCL9, DNTT, IL18R1, SOCS1, STAT2, CSF3R, FAS, PIK3R5, EBI3, MAP3K8, ACVR1B, PF4, CD44* |
| APICAL JUNCTION | 0.47 | 1.54 | 0.00 | 0.03 | *EXOC4, NECTIN4, COL17A1, NFASC, MAP3K20, CLDN4, ACTA1, PPP2R2C, CDH4, PARD6G, CLDN14, LIMA1, GRB7, VCL, BAIAP2, ICAM5, THY1, MAP4K2, CDH3, LAMB3* |
| ESTROGEN RESPONSE LATE | 0.47 | 1.53 | 0.00 | 0.03 | *PKP3, DCXR, TRIM29, TJP3, RAPGEFL1, OVOL2, SFN, ASS1, CLIC3, ST14, CA2, CACNA2D2, TOB1, SCNN1A, MAPT, PERP, DUSP2, TFAP2C, CELSR2, PGR* |
| COMPLEMENT | 0.46 | 1.52 | 0.00 | 0.03 | *TMPRSS6, ADRA2B, CDK5R1, CA2, KLK1, MMP15, KYNU, IRF7, F3, PLEK, CASP7, PSMB9, GCA, LCP2, F7, ITIH1, CFB, ACTN2, ITGAM, TFPI2* |
| COAGULATION | 0.46 | 1.47 | 0.02 | 0.04 | *C8A, MST1, TMPRSS6, MMP15, F3, PLEK, CTSE, MMP2, ITIH1, F13B, HPN, CFB, P2RY1, TFPI2, C1QA, F10, KLK8, LEFTY2, WDR1, GP9* |
| ESTROGEN RESPONSE EARLY | 0.44 | 1.47 | 0.01 | 0.05 | *PMAIP1, ESRP2, TJP3, RAPGEFL1, OVOL2, SFN, CLIC3, SLC19A2, SYT12, TOB1, SCNN1A, MAPT, ELF3, TFAP2C, CELSR2, ADCY1, PGR, TTC39A, MUC1, FOS* |

ES - Enrichment Score; NES - Normalized Enrichment Score; NOM p-val - Nominal p-value; FDR q-val - False discovery rate

**Supplementary Table S4. Primers used in this study.**

| **RT-qPCR Primers** | | |
| --- | --- | --- |
| **Gene** | **Sequence** | **Direction** |
| *Ankrd65* | AGTGGCTAAGGGCATTGAAATA | Forward |
| *Ankrd65* | GGAGATGGCTGACAACTTCTAC | Reverse |
| *Cbr2* | GGTAGCCAGGGACATGATTAAC | Forward |
| *Cbr2* | GAGCTGTAGGTGATCAAGTTAGG | Reverse |
| *Cdkn1a* | TTAGGCAGCTCCAGTGGCAACC | Forward |
| *Cdkn1a* | ACCCCCACCACCACACACCATA | Reverse |
| *Fermt1* | CATGCAAATGGAGAGCAGCAG | Forward |
| *Fermt1* | TTCCCACCACAGAGCATAGTC | Reverse |
| *Fermt3* | ATGGAGGCTCAGGGAACAAA | Forward |
| *Fermt3* | CTTGGCCTTGAACTTTCGCT | Reverse |
| *Gapdh* | TCCATGACAACTTTGGCATTG | Forward |
| *Gapdh* | CAGTCTTCTGGGTGGCAGTGA | Reverse |
| *Ddx4* | GTGGAAATACTGGCAGAGCG | Forward |
| *Ddx4* | ATCCTGTTGAGCGTCTGACA | Reverse |

| **Genotyping primers** | | | |
| --- | --- | --- | --- |
| **Gene** | **Probe** | **Sequence** | **Direction** |
| *Chek2* | *wild-type* | CTTGTCCTGCTGGACTCACA | Forward |
| *Chek2* | *wild-type* | GCAACCGTTACCTACCCTGA | Reverse |
| *Chek2* | *mutant* | CGGTCGCTACCATTACCAGT | Forward |
| *Chek2* | *mutant* | CAGCGCTTATCCCAACACT | Reverse |
| *Trp53* | *wild-type* | CAGCCTCTGTTCCACATACACT | Forward |
| *Trp53* | *mutant* | AGGCTTAGAGGTGCAAGCTG | Forward |
| *Trp53* | *wild-type and mutant* | TGGATGGTGGTATACTCAGAGC | Reverse |
| *Trp63* | *wild-type and mutant* | ACCTGGCTTCCTTCTCATTG | Forward |
| *Trp63* | *wild-type and mutant* | CTTTGATACGCTGCTGCTTG | Reverse |

## **Supplementary Data Files.**

Supplementary Data 1: Bulk RNAseq_Ovary_Gene_Counts_ DEGs_groupwise

Supplementary Data 2: scRNAseq_Ovary_Clusters_Marker_Genes_DEGs_groupwise

Supplementary Data 3: scRNAseq_Oocyte_Subclusters_Marker_Genes_DEGs

Supplementary Data 4: scRNAseq_Granulosa_Subclusters_Marker_Genes_DEGs
